# Supplementary material for: Neoadjuvant camrelizumab and apatinib combined with chemotherapy versus chemotherapy alone for locally advanced gastric cancer: a multicenter randomized phase 2 trial
Source: Nat Commun. 2024 Jan 2;15:41. doi: 10.1038/s41467-023-44309-5 (PMC10762218; doi:10.1038/s41467-023-44309-5)
Supplement: Supplementary file 1 — Supplementary Information [file 41467_2023_44309_MOESM1_ESM.pdf]

Supplementary Table 1. Efficacy analysis according to PD-L1 expression (CPS).

Variable	CA-SAP group	SAP group	P value
CPS <1%	n = 22	n = 23	
Major pathological response rate	27.3 (7.1-47.5)	17.4 (0.6-34.2)	0.331
Complete response rate	13.6 (0-29.2)	8.7 (0-21.2)	0.478
Objective response rate	68.2 (47.0-89.3)	43.5 (21.6-65.4)	0.086
R0 resection rate	95.5 (86.0-100)	87.0 (72.1-100)	0.321
CPS ≥1%	n = 27	n = 27	
Major pathological response rate	40.7 (20.9-60.5)	18.5 (2.9-34.2)	0.068
Complete response rate	18.5 (2.9-34.2)	3.7 (0-11.3)	0.096
Objective response rate	66.7 (47.7-85.7)	44.4 (24.4-64.5)	0.085
R0 resection rate	100 (NA)	85.2 (70.9-99.5)	0.056
CPS ≥5%	n = 16	n = 11	
Major pathological response rate	50.0 (22.5-77.5)	27.3 (0-58.7)	0.107
Complete response rate	18.8 (0-40.2)	9.1 (0-29.3)	0.455
Objective response rate	68.8 (43.2-94.3)	27.3 (0-58.7)	0.193
R0 resection rate	100 (NA)	81.8 (54.6-100)	0.157

NOTE: Data are percentages and 95% confidence intervals. P values were one-sided in Fisher's exact test. No adjustments were made for multiple comparisons. CA-SAP = camrelizumab, apatinib, nap-paclitaxel, and S-1; SAP = nap-paclitaxel and S-1; NA = not applicable.

Supplementary Table 2. Treatment-related adverse events with potential immunological cause during neoadjuvant therapy.

Adverse Events	CA-SAP group (n=51)	SAP group (n=53)
	Any Grade	Any Grade
Hypothyroidism	7 (13.7)	1 (1.9)
Hyperthyroidism	0 (0.0)	0 (0.0)
Immune pneumonitis	2 (3.9)	0 (0.0)
Colitis	0 (0.0)	0 (0.0)
Hepatitis	2 (3.9)	0 (0.0)
Nephritis	0 (0.0)	0 (0.0)
Pancreatitis	0 (0.0)	0 (0.0)
Severe skin reactions	0 (0.0)	0 (0.0)
Adrenal insufficiency	0 (0.0)	0 (0.0)
Type 1 diabetes	0 (0.0)	0 (0.0)
Hypophysitis	1 (2.0)	0 (0.0)

NOTE. Data are No. (%).

Supplementary Table 3. Surgical outcomes.

Variable	CA-SAP group (n=49)	SAP group (n=49)	P value
Postoperative recovery			
Time to ambulation (days)	2 (1-2)	2 (1-2)	0.910
Time to first flatus (days)	3 (2-3)	3 (2-3)	0.126
Time to first liquid intake (days)	4 (3-4)	3 (3-4)	0.105
Postoperative hospital stay (days)	8 (7-11)	8 (7-9)	0.592
Postoperative morbidity	10 (20.4)	6 (12.2)	0.295
Anastomotic leakage	4 (8.2)	1 (2.0)	0.201
Gastroplegia	1 (2.0)	0 (0.0)	1.000
Pneumonia	4 (8.2)	5 (10.2)	1.000
Pneumothorax	1 (2.0)	0 (0.0)	1.000
Major complications	1 (2.0)	1 (2.0)	1.000
Postoperative transfusion	3 (6.1)	3 (6.1)	1.000
Reoperation	0 (0.0)	0 (0.0)	1.000
Unplanned readmission	2 (4.1)	1 (2.0)	1.000
In-hospital or 30-day mortality	0 (0.0)	0 (0.0)	1.000

NOTE: Data are No. (%) or median (first quartile-third quartile [Q1-Q3]). P values were two-sided in the Wilcoxon rank sum test, χ^2 test or Fisher's exact test. No adjustments were made for multiple comparisons. CA-SAP = camrelizumab, apatinib, nap-paclitaxel, and S-1; SAP = nap-paclitaxel and S-1.

Supplementary Table 4. Clinicopathological Characteristics of the Historical Cohort.

Variable	C-SAP cohort	P value[a]	P value[b]
Age, years[c]	60 (54-68)	0.220	0.367
Sex[c]			
Male	30 (66.7)	0.077	0.948
Female	15 (33.3)		
ECOG performance status[c]			
0	28 (62.2)	0.801	0.555
1	17 (37.8)		
Lauren classification[c]			
Intestinal	14 (31.1)	0.407	0.253
Diffuse	29 (64.4)		
Unknown	2 (4.4)		
Tumor location[c]			
Upper 1/3	18 (40.0)	0.958	0.389
Middle 1/3	10 (22.2)		
Lower 1/3	11 (24.4)		
Mixed	6 (13.3)		
Tumor size, mm[c]	58 (45-70)	0.130	0.270
Borrmann type[c]			
II-III	41 (91.1)	0.315	0.926
IV	4 (9.9)		
cT stage[c]			
T3	3 (6.7)	0.579	0.188
T4	42 (93.3)		
Type of gastrectomy[d]			
Total	31 (77.5)	0.811	0.113
Distal	9 (22.5)		
Lymphovascular invasion[d]			
No	22 (55.0)	0.708	0.839
Yes	18 (45.0)		
Neural invasion[d]			
No	23 (57.5)	0.169	0.078
Yes	17 (42.5)		
ypT stage[d]			
T0	3 (6.7)	0.521	0.882
T1	7 (15.6)		
T2	4 (10.0)		
T3	17 (42.5)		
T4a	9 (22.5)		
ypN stage[d]			
N0	16 (40.0)	0.527	0.791
N1	5 (12.5)		
N2	9 (22.5)		
N3	10 (25.0)		
ypM stage[d]			
M0	40 (100.0)	1.000	0.500
M1	0 (0.0)		

[a] Characteristics were compared between the C-SAP cohort and the CA-SAP group.

[b] Characteristics were compared between the C-SAP cohort and the SAP group.

[c] Characteristics were compared between the whole C-SAP cohort (n=45) and the modified intention-to-treat sets (CA-SAP: n=51; SAP: n=53).

[d] Characteristics were compared between the C-SAP cohort proceeding to gastrectomy (n=40) and the per-protocol sets (CA-SAP: n=49; SAP: n=49).

NOTE: Data are No. (%) or median (first quartile-third quartile [Q1-Q3]). Because of rounding, not all percentages add up to 100%. P values were two-sided in the Wilcoxon rank sum test, χ^2 test or Fisher's exact test. No adjustments were made for multiple comparisons. C-SAP = camrelizumab, nap-paclitaxel, and S-1; CA-SAP = camrelizumab, apatinib, nap-paclitaxel, and S-1; SAP = nap-paclitaxel and S-1; ECOG = Eastern Cooperative Oncology Group.

Supplementary Table 5. Efficacy analysis of the Historical Cohort.

Variable	C-SAP cohort (n=45)	CA-SAP group (n=51)	SAP group (n=53)
Major pathological response rate	24.4 (11.4-37.5)	33.3 (19.9-46.7)	17.0 (6.5-27.4)
Complete response rate	6.7 (0-14.2)	15.7 (5.4-26.0)	5.7 (0-12.1)
Objective response rate	51.1 (35.9-66.3)	66.0 (52.4-79.6)	43.4 (29.2-57.6)
R0 resection rate	88.9 (79.3-98.4)	94.1 (87.4–100)	81.1 (70.2–92.0)

NOTE: Data are percentages and 95% confidence intervals. C-SAP = camrelizumab, nap-paclitaxel, and S-1; CA-SAP = camrelizumab, apatinib, nap-paclitaxel, and S-1; SAP = nap-paclitaxel and S-1.

Supplementary Table 6. Eligibility criteria for enrolling patients.

Inclusion
Age older than 18 and younger than 75 years
Primary gastric adenocarcinoma confirmed pathologically by endoscopic biopsy
Clinical stage T2-4aN+M0 disease as assessed by CT/MRI, PET-CT, and laparoscopy, if feasible
At least one measurable lesion according to the RECIST, version 1.1
ECOG performance status of 0 or 1
Life expectancy of at least 12 weeks
Acceptable bone marrow, hepatic, and renal function, including:
- White blood cell count >3.0 × 10^9 cells per L and platelet count >100 × 10^9 cells per L
- Total bilirubin <1.5 × ULN, alanine aminotransferase or aspartate amino transferase <2.5 × ULN
- Serum creatinine ≤1 × ULN, or calculated glomerular filtration rate >60 mL/min
Written informed consent
Exclusion
Prior chemotherapy, radiotherapy, targeted therapy or immunotherapy
Contraindications for surgical treatment or chemotherapy
Presence of distant metastasis
History of other malignant disease within the past 5 years, except: basal cell carcinoma of the skin, squamous cell carcinoma of the skin, or in situ cervical cancer
Any active or history of autoimmune disease, or history of syndrome that required systemic steroids or immunosuppressive medications, except for subjects with vitiligo or resolved childhood asthma/atopy
History of immunodeficiency diseases, including human immunodeficiency virus (HIV), or other acquired or congenital immune-deficient disease, or transplantation
Severe heart, lung, liver, or renal dysfunction
Severe mental disorder
Presence of digestive tract obstruction, jaundice, or acute infectious diseases
Pregnant or breast-feeding women
Uncontrolled blood pressure on medication (>140/90 mmHg)
Evidence of bleeding tendency or receiving thrombolytics or anticoagulants
History of interstitial lung disease or non-infectious pneumonia
Positive urinary protein (urinary protein ≥2+ or 24h urine protein >1 g)
Sensitivity to investigational drugs

CT = Computed tomography; MRI = Magnetic resonance imaging; PET = Positron emission tomography; RECIST = Response Evaluation Criteria in Solid Tumors; ULN = Upper limits of normal; ECOG = Eastern Cooperative Oncology Group.

Supplementary Figure 1. Design of the Arise-FJ-G005 study. Treatment processes of two patients who were treated with CA-SAP (Patient A shown in orange) and SAP (Patient B shown in blue) are provided. Scale bar = 100 μm. CA-SAP = camrelizumab, apatinib, nap-paclitaxel, and S-1; SAP = nap-paclitaxel and S-1; PD-L1 = programmed death-ligand 1; CPS = combined positive score; IHC = immunohistochemistry; CT = computed tomography.

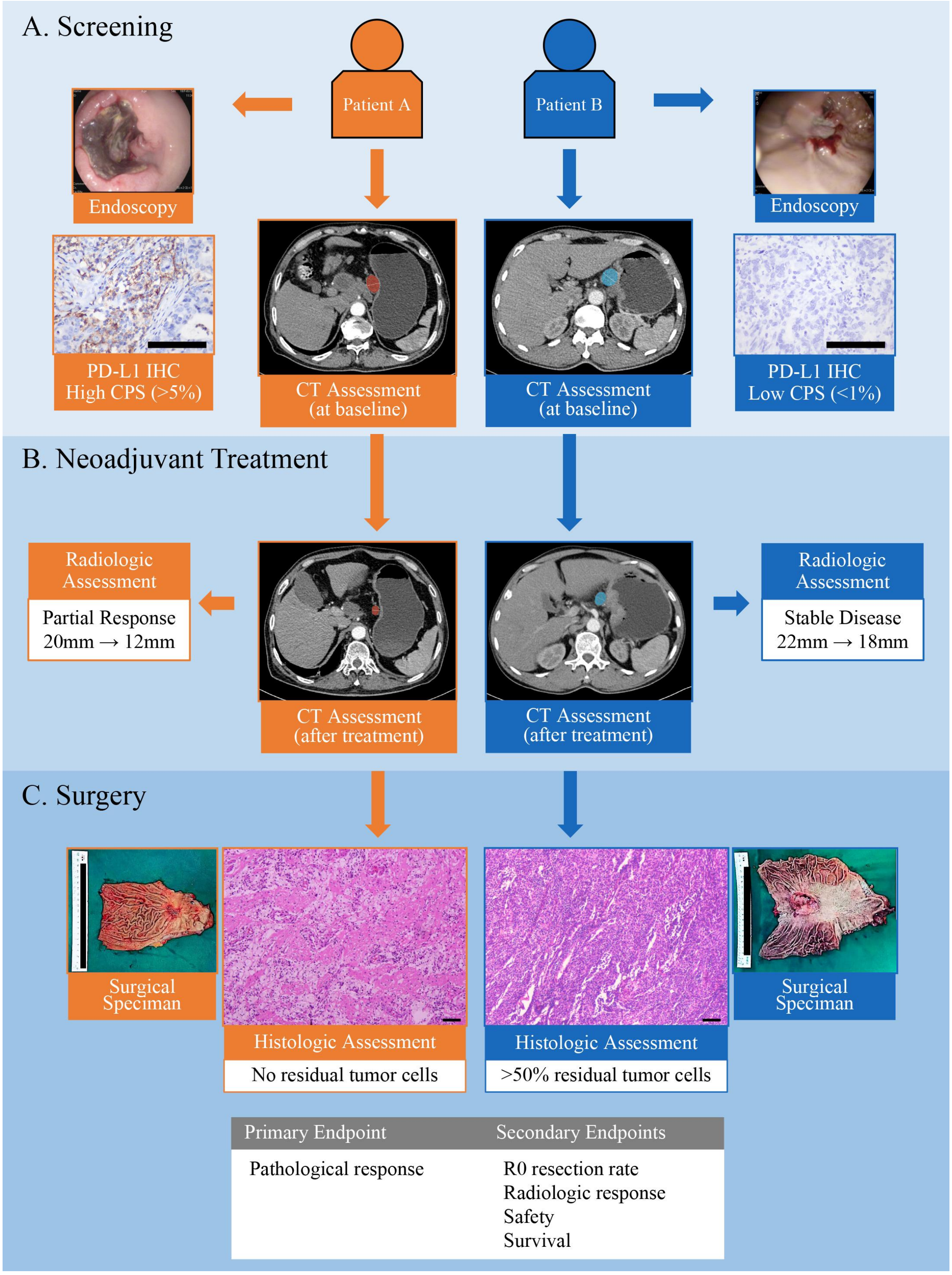

Supplementary Note 1

Data Management and Sharing Plan of Fujian Medical University Union Hospital

1. **Purpose**

This policy aims to ensure that the hospital's data management and sharing practices comply with relevant regulations, protect patient privacy, and promote effective data management and sharing.

2. **Scope**

This policy applies to all departments and personnel within the hospital and encompasses all data related to the hospital.

3. **Data Classification**

The hospital's data will be categorized based on sensitivity and shareability into the following types:

Sensitive Patient Data: Includes patient diagnoses, medical records, identity information, etc.
Medical Research Data: Involves data related to medical research and clinical trials.
Administrative Data: Covers data related to hospital operations, finances, and human resources.

Public Data: Non-sensitive data that can be made publicly accessible.

4. **Data Collection and Storage**

The hospital will take appropriate measures to ensure secure data collection, storage, and backup. This includes encryption, access controls, and regular reviews.

5. **Data Sharing**

Data sharing must adhere to applicable regulations and legal requirements. When sharing data, patient or research subject consent (if required) must be obtained, and data must be transmitted in a secure manner.

6. **Data Protection and Privacy**

The hospital will implement measures to ensure the privacy and security of patient data. This includes data access controls, staff training, and an incident response plan.

7. **Data Management Team**

The hospital will establish a data management team responsible for developing and implementing data management and sharing plans. This team will conduct regular policy

reviews and updates.

8. Review and Updates

This policy will undergo periodic reviews to ensure alignment with regulations and actual needs and will be updated as necessary.

9. Compliance and Oversight

The hospital will maintain compliance and oversight of data management and sharing practices to ensure policy adherence and implementation.

10. Education and Training

The hospital will provide training on data management and sharing policy to ensure all staff members are aware of and comply with the policy. Please note that this is just a sample template, and specific policy content and requirements may vary based on the hospital's specific circumstances and regulatory requirements. When creating policies, it is advisable to consult legal counsel and data protection experts to ensure policy legality and practicality.

Supplementary Note 2

Study Protocol (v1.0, v1.3, and summary of changes)

Efficacy and Safety of Camrelizumab and Apatinib Combined with Nab-paclitaxel and S-1 as Neoadjuvant Treatment for Locally Advanced Gastric Cancer: A Multicenter Randomized Controlled Trial

CLINICAL STUDY PROTOCOL

Registration Number: NCT04195828

Principle Investigator: Chang-Ming Huang, Ph.D.
Department of Gastric Surgery, Fujian Medical University Union Hospital, No. 29 Xinquan Road, Fuzhou, Fujian Province, China.
Telephone: +86-591-83363366
E-mail: hcmlr2002@163.com

Coordinating Investigator: Jian-Xin Ye, Ph.D.
First Affiliated Hospital of Fujian Medical University
E-mail: yjx0201@vip.sina.com

Kai Ye, Ph.D.
Second Affiliated Hospital of Fujian Medical University
E-mail: Yekai1972@126.com

Wei-Dong Zang, Ph.D.
Fujian Provincial Cancer Hospital
E-mail: 894434459@qq.com

Jian-Chun Cai, Ph.D.
Zhongshan Hospital of Xiamen University
E-mail: jianchunfh2@sina.com

Jun You, Ph.D.
First Affiliated Hospital of Xiamen University

E-mail: youjunxm@163.com

Li-Sheng Cai, Ph.D.
Zhangzhou Municipal Hospital of Fujian Province
E-mail: 1272110762@qq.com

Yan-Chang Xu, Ph.D.
The First Hospital of Putian
E-mail: cccxyc@163.com

Wei Lin, Ph.D.
The Affiliated Hospital of Putian University
E-mail: linwbj@outlook.com

TABLE OF CONTENTS

Abbreviations ... 1
SYNOPSIS ... 3
Study Plan ... 8
1. Background ... 10
2. Objectives ... 13
    2.1 Primary Objective ... 13
    2.2 Secondary Objectives ... 13
    2.3 Exploratory Objective ... 13
3. Study Design and Sample Size ... 14
    3.1 Study Design ... 14
    3.2 Study Schema ... 14
    3.3 Study Duration ... 14
    3.4 Sample Size ... 14
4. Study Population ... 15
    4.1 Inclusion Criteria ... 15
    4.2 Exclusion Criteria ... 15
    4.3 Drop Out/Removal Criteria ... 16
    4.4 Discontinue Criteria ... 16
5. Study Treatments ... 17
    5.1 Handling and Storage ... 17
    5.2 Treatment ... 17
    5.3 Dose Modification ... 18
        5.3.1 Dose Modification for Camrelizumab ... 18
        5.3.2 Dose Modification for Apatinib ... 21
        5.3.3 Dose Modification for Chemotherapeutic Agents ... 23
    5.4 Concomitant Therapy ... 25
    5.5 Prudently or Forbidden Used Drug ... 25
6. Study Procedure ... 26
    6.1 Screening Period ... 26
    6.2 Treatment Period ... 26
        6.2.1 Neoadjuvant Treatment Period ... 27
        6.2.2 Preoperative Assessments and Surgery ... 27
        6.2.3 Adjuvant Treatment Period ... 27
    6.3 Follow-up Period ... 28
    6.4 Progression of Disease ... 28
7. Efficacy Assessments ... 29
8. Safety Assessments ... 30
    8.1 Safety Endpoints ... 30
    8.2 Definition of Adverse Event and Serious Adverse Event ... 30
    8.3 Recording and Follow-up of (Serious) Adverse Events ... 31
9. Statistical Analysis Plan ... 32
    9.1 Sample Size Considerations ... 32

9.2 Analysis Sets 32
9.3 Statistical Analysis 33
10. Data Management 33
10.1 CRF Completion Guidelines 33
10.2 Data Lock 34
10.3 Quality Control and Quality Assurance 34
11. Ethics and Regulatory Considerations 34
11.1 Local Regulations / Helsinki Declaration 34
11.2 Informed Consent 35
11.3 Independent Ethics Committee/Institutional Review Board 35
11.4 Confidentiality Agreement 35
11.5 Record Storage 35
11.6 Study Interruption 36
11.7 Protocol Modification 36
References 37
Appendix 1 Becker Regression Criteria 40
Appendix 2 Pathological Staging System 41
Appendix 3 Surgical Manual 43
Appendix 4 Response Evaluation Criteria in Solid Tumors (RECIST), Version 1.1 45
Appendix 5 ECOG Performance Status 47
Appendix 6 Classification of Surgical Complications 48

Abbreviations

Abbreviations	Full names
AE	Adverse event
ALT	Alanine aminotransferase
ALP	Alkaline phosphatase
ANC	Absolute neutrophil count
APTT	Activated partial thromboplastin time
AST	Aspartate aminotransferase
BSA	Body surface area
BUN	Blood urea nitrogen
CA	Carbohydrate antigen
CEA	Carcinoembryonic antigen
CI	Confidence interval
CR	Complete response
CRF	Case Report Form
CT	Computed tomography
CTCAE	Common Terminology Criteria for Adverse Events
DFS	Disease-free survival
DRQ	Data Request
ECG	Electrocardiography
ECOG	Eastern Cooperative Oncology Group
GC	Gastric cancer
HBV	Hepatitis B virus
HCV	Hepatitis C virus
HIV	Human immunodeficiency virus
IEC	Independent Ethics Committee
IQR	Interquartile range
IRB	Institutional Review Board

Abbreviations	Full names
irAE	Immune-related adverse event
ITT	Intention-to-treat
LAGC	Locally advanced gastric cancer
MPR	Major pathological response
MRI	Magnetic resonance imaging
NMPA	National Medical Products Administration
OB	Occult blood
ORR	Objective response rate
OS	Overall survival
PD	Progression disease
PD-1	Programmed cell death protein 1
PD-L1	Programmed cell death protein-ligand 1
PET	Positron emission tomography
PFS	Progression-free survival
PR	Partial response
PT	Prothrombin time
RBC	Red blood cell
RCCEP	Reactive cutaneous capillary endothelial proliferation
SAE	Serious adverse event
SD	Stable disease
TRG	Tumor regression grade
TSH	Thyroid-stimulating hormone
TT	Thrombin time
RECIST	Response Evaluation Criteria in Solid Tumors
ULN	Upper limits of normal
VEGF	Vascular endothelial growth factor
WBC	White blood cell

SYNOPSIS

**Title**	Efficacy and Safety of Camrelizumab and Apatinib Combined with Nab-paclitaxel and S-1 as Neoadjuvant Treatment for Locally Advanced Gastric Cancer:<br>A Multicenter Randomized Controlled Trial
**Study Subjects**	Patients with locally advanced gastric cancer (LAGC)
**Study Design**	Multicenter, open-label, randomized phase 2 clinical trial
**Study Objectives**	**Primary endpoint**<br>➢ Major pathological response (MPR)<br>**Secondary endpoints**<br>➢ Radiologic response according to the Response Evaluation Criteria in Solid Tumors (RECIST), version 1.1<br>➢ R0 resection rate<br>➢ Safety of study drugs according to the Common Terminology Criteria for Adverse Events (CTCAE), version 5.0<br>➢ Surgical safety: including morbidity, mortality, hospital stay, etc.<br>➢ Overall survival (OS), Progression-free survival (PFS)
**Number of Patients**	53 patients per group
**Inclusion Criteria**	● Age older than 18 and younger than 75 years<br>● Primary gastric adenocarcinoma confirmed pathologically by endoscopic biopsy<br>● Clinical stage T2-4aN+M0 disease as assessed by CT/MRI, PET-CT, and laparoscopy, if feasible<br>● At least one measurable lesion according to the RECIST, version 1.1<br>● Eastern Cooperative Oncology Group (ECOG) performance status of 0 or 1<br>● Life expectancy of at least 12 weeks<br>● Acceptable bone marrow, hepatic, and renal function, including:

	blood routine examination(No blood transfusion within 14 days)<br>1. White blood cell count >3.0 × 10^9 cells per L and platelet count >100 × 10^9 cells per L<br>2. Total bilirubin <1.5 × ULN, alanine aminotransferase or aspartate amino transferase <2.5 × ULN<br>3. Serum creatinine ≤1 × ULN, or calculated glomerular filtration rate >60 mL/min<br>● Written informed consent
**Exclusion Criteria**	● Prior chemotherapy, radiotherapy, targeted therapy, or immunotherapy<br>● Contraindications for surgical treatment or chemotherapy<br>● Presence of distant metastasis<br>● History of other malignant disease within the past 5 years, except: basal cell carcinoma of the skin, squamous cell carcinoma of the skin, or in situ cervical cancer<br>● Any active or history of autoimmune disease, or history of syndrome that required systemic steroids or immunosuppressive medications, except for subjects with vitiligo or resolved childhood asthma/atopy<br>● History of immunodeficiency diseases, including human immunodeficiency virus (HIV), or other acquired or congenital immune-deficient disease, or transplantation<br>● Severe heart, lung, liver, or renal dysfunction<br>● Severe mental disorder<br>● Presence of digestive tract obstruction, jaundice, or acute infectious diseases<br>● Pregnant or breast-feeding women<br>● Uncontrolled blood pressure on medication (>140/90 mmHg)<br>● Evidence of bleeding tendency or receiving thrombolytics or anticoagulants<br>● History of interstitial lung disease or non-infectious pneumonia<br>● Positive urinary protein (urinary protein≥2+ or 24h urine

	protein >1 g)<br>● Sensitivity to study drugs
**Drop out/removal criteria**	● Use of NMPA-approved modern traditional Chinese medicine agents for the treatment of gastric cancer<br>● Concurrent radiotherapy or other local treatments during the study periods<br>● Medication, dosing, and treatment methods do not follow the study protocol
**Discontinue Criteria**	● Withdrawn of the informed consent<br>● Progression disease (PD)<br>● Unbearable toxicity after dose reductions<br>● Pregnancy<br>● Loss to follow up<br>● Investigator's decision that stopping treatment was in the best interest of the patient<br>● Death
**Study Duration**	● Planned start date: November 1, 2019<br>● Planned enrollment completion date: October 31, 2021<br>● Planned study end date: October 31, 2024
**Treatment Regimens**	**Neoadjuvant treatment**<br>**Treatment group (CA-SAP):**<br>● Camrelizumab, 200 mg intravenously on day 1;<br>● Apatinib, 250 mg orally once daily on days 1 to 21;<br>● Nap-paclitaxel, 125 mg/m^2 intravenously on days 2 and 9;<br>● S-1 (Tigio), 40-60 mg orally twice daily on days 1 to 14.<br>**Control group (SAP):**<br>● Nap-paclitaxel, 125 mg/m^2 intravenously on days 1 and 8;<br>● S-1 (Tigio), 40-60 mg orally twice daily on days 1 to 14.<br>The dose of S-1 is based on body surface area (BSA):<br>BSA | Dose

<table>
<tr>
<td></td>
<td>
<table>
<tr><td>< 1.25m²</td><td>40mg× 2/day</td></tr>
<tr><td>1.25m² - 1.5m²</td><td>50mg× 2/day</td></tr>
<tr><td>> 1.5m²</td><td>60mg× 2/day</td></tr>
</table>
The above treatments will be administered every 3 weeks for three preoperative cycles.
Surgery
Surgery was scheduled 2 to 4 weeks after completion of the last cycle of neoadjuvant treatment. All surgical procedures, including the extent of gastric resection and D2 lymph node dissection, are performed according to the guidelines of the Japanese Research Society for the Study of Gastric Cancer.
Adjuvant treatment
Adjuvant treatment started 3 to 8 weeks after gastrectomy. Patients will receive five 3-week cycles of adjuvant treatment with CA-SAP or SAP according to the preoperative regimen.
</td>
</tr>
<tr>
<td>Statistical Consideration</td>
<td>
Analysis sets
<u>Intention-to-treat (ITT)</u> set included all patients who are randomly assigned. This population will be used for the efficacy analyses.
<u>Per-protocol</u> set included patients in the ITT population who did not present major deviations from the protocol. We pre-specified that participants who received surgery after administration of neoadjuvant CA-SAP or SAP are included in this set. This population will be also used for the efficacy analyses.
<u>Safety analysis set</u> included patients who received at least one dose of allocated treatment. This population will be also used for the safety analyses.
Sample size determination
Based on the assumption of MPR rates of 15% in the SAP group and 35% in the CA-SAP group, a sample size of 53 patients per group
</td>
</tr>
</table>

	was required to detect improvement with 80% power and a one-sided alpha level of 0.1 (Fisher's exact test), including a 5% dropout rate.
**Version**	1.0

Study Plan

Items	Screening Period		Randomization	Neoadjuvant Treatment Period					Surgery		Adjuvant Treatment Period		Follow-up[15]
	Before 2 weeks of enrollment	Before 1 week of enrollment		Cycle 1	Cycle 2		Cycle 3		Before 1 week of surgery	After 1 week of surgery	Cycle 1-5		
				Day 8 (±3)	Day 1 (±3)	Day 8 (±3)	Day 1 (±3)	Day 8 (±3)			Day 1 (±3)	Day 8 (±3)	
**Baseline characters**													
**Informed consent**	Prior to randomization												
**Demographics data**	×												
**Medical history**	×												
**Vital signs and physical**		×		×	×	×	×	×	×	×	×	×	×
**Assessments**													
**Blood routine[2]**		×		×	×	×	×	×	×	×	×	×	×
**Urinary routine[3]**		×			×		×		×		×		
**Fecal routine[4]**		×			×		×		×		×		
**Biochemistry[5]**		×		×	×	×	×	×	×	×	×	×	×
**Coagulation test[6]**		×			×		×		×		×		
**Thyroid function test[7]**		×			×		×		×		×		
**Tumor markers[8]**		×			×		×		×		×		×
**HBV/HCV/HIV[9]**		×							×				
**Tumor sample[10]**		×								×			
**Gastroduodenoscopy**		×											
**12-lead ECG**		×			×		×		×		×		
**Pregnancy test[11]**		×											

**Imaging examination[12]**	×						×		×				×
**ECOG status**		×			×		×		×	×	×		
**Blood pressure monitoring[13]**		×		×	×	×	×	×	×	×	×	×	
**Adverse events[14]**				×	×	×	×	×	×	×	×	×	
**Others**													
**Concomitant treatments**	×			×	×	×	×	×	×	×	×	×	
**Drug compliance**					×		×		×		×		

[1] Including vital signs (blood pressure, pulse, and temperature), height (at baseline only), weight, and physical examination (especially abdominal examination).

[2] Including hemoglobin, hematocrit, RBC, WBC, ANC, and platelet count.

[3] Including urinary protein and urine occult blood.

[4] Including fecal occult blood. Gastroduodenoscopy should be performed if the patient has persistent OB (+) before resection of primary tumor.

[5] Including bilirubin, aspartate aminotransferase (AST), alanine aminotransferase (ALT), alkaline phosphatase (ALP), creatinine, blood urea nitrogen (BUN), glucose, calcemia, sodium, and potassium.

[6] Including prothrombin time (PT), activated partial thromboplastin time (APTT), thrombin time (TT), and fibrinogen.

[7] Including thyroid-stimulating hormone (TSH), free T3, and free T4.

[8] Including carcinoembryonic antigen (CEA) and carbohydrate antigen (CA)19-9.

[9] Including hepatitis B virus (HBV), hepatitis C virus (HCV), and human immunodeficiency virus (HIV).

[10] Central evaluation of programmed death-ligand 1 (PD-L1) expression and microsatellite instability (MSI) in pretreatment specimens, and tumor regression grade in surgical specimens.

[11] For women of child-bearing age.

[12] Including chest, abdomen and pelvis CT or MRI.

[13] Blood pressure monitoring is completed by the patient and recorded in a diary card. Blood pressure should be measured at least 3 times a week for the first 2 cycles. For patients with abnormal blood pressure, blood pressure should be measured every day. At each visit, blood pressure should be measured by the investigator. Each blood pressure measurement should be taken ipsilateral.

[14] Adverse events are recorded from randomization until 30 days after the last dose of the investigational product. During follow-up, all adverse events that occur previously and are ongoing, and newly occurring adverse events related with the investigational product or determined by the investigator as having a reasonable possibility of being caused by the investigational product are recorded, and of these, serious adverse events are all reported. Serious adverse events ongoing at the time of completion of the investigational product administration will be followed during the follow-up period until resolved or stabilized irrespective of the relationship with the investigational product.

[15] Patients are followed up postoperatively by physical examination, laboratory tests, and imaging examination every 3 months for 2 years, every 6 months during years 3-5, and annually thereafter. Endoscopy is recommended annually.

1. Background

There were over 1,000,000 new cases and an estimated 783,000 deaths caused by gastric cancer (GC) in 2018, making it the fifth most frequently diagnosed cancer and the third leading cause of cancer death [1]. It is a serious threat to human health and brings a heavy economic burden to families and society. As the National Central Cancer Registry reported, there were approximately 410,000 new cases of GC and 290,000 deaths every year in China [2]. With the improvement of diagnosis and treatment methods and deepening understanding of the molecular mechanism, the incidence and mortality of GC have shown a downward trend. However, it still faces great challenges.

Endoscopic or surgical resection is curative in most early gastric cancers, with a 5-year overall survival (OS) rate greater than 90% [2]. However, there are no specific signs with early GC, and most patients are diagnosed at advanced stages at first admission [3]. Compared with early GC, advanced GC often causes invasion and adhesion to surrounding tissues and sheds tumor cells into the blood, leading to a low radical resection rate [4]. Disease recurrence occurs in over 50% of patients even after the complete dissection of the primary tumors and regional lymph nodes due to micrometastases [3]. To improve the prognosis of patients with advanced GC, the treatment mode has changed from “surgery alone” to “neoadjuvant chemotherapy + surgery + adjuvant chemotherapy” [4].

Neoadjuvant chemotherapy was first introduced in the multimodal treatment of GC in 1989 by Wilke et al. [5]. In this study, advanced GC patients were treated with EAP regimen. Among them, 33 patients underwent R0/R1 surgical resection and postoperative chemotherapy, with a response (CR+PR) rate of 70%. A meta-analysis by Paoletti et al. showed that the 5-year survival rate was improved by 5.7% in patients undergoing neoadjuvant chemotherapy plus surgery than in those undergoing surgery alone [6]. The MAGIC phase 3 trial published in 2006 demonstrated that perioperative

chemotherapy with epirubicin, cisplatin, and fluorouracil (ECF) could increase the 5-year OS rate from 23% to 36% compared with surgery alone [7]. The FNCLCC and FFCD trial also demonstrated that perioperative chemotherapy using fluorouracil plus cisplatin significantly increased the R0 resection rate, disease-free survival (DFS), and OS [8]. In the FLOT4 trial published in 2019, OS and DFS were significantly improved in the FLOT group (fluorouracil, leucovorin, oxaliplatin, and docetaxel) compared with the ECF/ECX group (epirubicin, cisplatin, and fluorouracil) [9]. Based on these results, the FLOT has become a new standard perioperative regimen in Europe. In China, neoadjuvant chemotherapy using platinum and fluorouracil with or without paclitaxel has been recommended as the first-line treatment for LAGC [10].

Although neoadjuvant chemotherapy has been widely accepted in both Eastern and Western countries, there is still no consensus on the optimal chemotherapy regimen. In recent years, paclitaxel-based chemotherapy has proven efficacy in GC [9,11-12]. Nanoparticle albumin bound (nab)-paclitaxel is a 130 nm particle formulation consisting of paclitaxel and albumin nanoparticles linked by a non-covalent bond, which minimizes the risk of hypersensitivity reactions without premedication. Additionally, nab-paclitaxel exhibits distinct biodistribution properties and increased antitumor efficacy compared with solvent-based paclitaxel [13-14]. In vitro, nab-paclitaxel showed stronger antitumor activity on human gastric cancer cell lines than oxaliplatin and epirubicin [15].

In the past decade, several clinical trials have been performed to investigate molecular targeted therapy for GC, but few molecular agents have shown promising activity [16-18]. The ToGA trial demonstrated that trastuzumab with cisplatin and capecitabine or 5-FU (XP/FP) was associated with improved OS for HER2-positive advanced GC [19]. Unfortunately, only a small percentage of patients (approximately 15%) are ideal candidates for HER-2 targeted therapy. Another well-established target is vascular endothelial growth factor (VEGF). VEGF is one of the most potent angiogenic factors and is a signaling molecule secreted by many solid tumors [20]. Since high VEGF expression is one of the characteristic features of gastric carcinomas, targeting VEGF is therefore considered a promising therapeutic strategy. Apatinib, a

novel receptor tyrosine kinase inhibitor selectively targeting VEGF receptor 2 (VEGFR-2), strongly inhibited VEGF-mediated endothelial cell migration, proliferation, and tumor microvascular density [21]. A phase III study showed that apatinib improved OS and PFS in patients with chemotherapy-refractory advanced or metastatic GC when compared with placebo [22]. In our earlier study, apatinib combined with chemotherapy was effective as neoadjuvant treatment for LAGC, with a MPR rate of 25.0%, and had an acceptable safety profile [23].

Host immunity is fundamental to the suppression of human cancer, and conversely host immune evasion by tumor cells is an essential feature in the development and progression of human cancer. Programmed cell death protein 1 (PD-1) is a co-inhibitory receptor expressed on the surface of activated and exhausted T-cells, B-cells, and certain myeloid cells [24]. PD-L1, one of two ligands for PD-1, is highly expressed in certain human tumors and expression has been associated with a poor prognosis [25-26]. PD-1 inhibitor, which suppresses the interaction between the PD-1 and its ligands, has demonstrated encouraging antitumor activity in advanced GC, particularly in combination with chemotherapy. In the KEYNOTE-061 study, pembrolizumab did not significantly improve OS or PDS compared with paclitaxel [27]. In contrast, pembrolizumab exhibited a higher ORR (60.0% vs. 25.8%) in combination with cisplatin and fluorouracil compared with its monotherapy in the KEYNOTE-059 study [28].

Multiple preclinical models have supported the synergistic effects between angiogenic inhibitors and PD-1 inhibitors [29-30]. In the IMmotion151 trial, atezolizumab (anti－PD-L1) + bevacizumab (anti-VEGF) showed longer PFS compared with sunitinib in patients with metastatic renal cell carcinoma [31]. These findings suggest a strong rationale for combining PD-1 inhibitors and angiogenic inhibitors. Camrelizumab, a high-affinity humanized IgG4 monoclonal antibody targeting PD-1, has shown clinically significant efficacy in advanced GC [32]. Thirty patients with chemotherapy-refractory recurrent or metastatic GC were enrolled. Of these, 7 patients (23.3%) demonstrated objective responses, including 1 CR. The ORRs for patients with PD-L1–positive and PD-L1–negative tumors were 23.1% (3 of 13) and

26.7% (4 of 15), respectively (P = 1.000). Two treatment-related grade 3 or higher adverse events were reported: one was grade 3 pruritus, and the other (3.3%) was grade 5 interstitial lung disease. We hypothesized that camrelizumab and apatinib combined with chemotherapy might be beneficial in patients with LAGC. Thus, we conducted a phase 2 trial (Arise-FJ-G005) to investigate the efficacy and safety of camrelizumab and apatinib combined with nab-paclitaxel plus S-1 versus nab-paclitaxel plus S-1 alone as neoadjuvant treatment for LAGC.

2. Objectives

2.1 Primary Objective

To assess the efficacy of camrelizumab and apatinib combined with nab-paclitaxel plus S-1 versus nab-paclitaxel plus S-1 alone as neoadjuvant treatment for LAGC, as measured by MPR according to the Becker regression criteria.

2.2 Secondary Objectives

To assess and compare the followings between the two groups:

- Radiologic response according to the RECIST, version 1.1
- R0 resection rate
- Safety of study drugs according to the CTCAE, version 5.0
- Surgical safety: including morbidity, mortality, hospital stay, etc.
- OS and PFS

2.3 Exploratory Objective

To assess the associations between the primary endpoint and several tumor biomarkers (including but not limited to PD-L1 expression and MSI status)

3. Study Design and Sample Size

3.1 Study Design

This is a multicenter, randomized, open-label, phase 2 trial to investigate the efficacy and safety of camrelizumab and apatinib combined with nab-paclitaxel plus S-1 versus nab-paclitaxel plus S-1 alone as neoadjuvant treatment for LAGC. It will be conducted in 5 medical centers in China. Eligible patients were randomly assigned to receive camrelizumab (200 mg intravenously on day 1) and apatinib (250 mg orally once daily on days 1 to 21) combined with chemotherapy (nap-paclitaxel 125 mg/m^2 intravenously on days 2 and 9, S-1 40 to 60 mg orally twice daily depending on BSA on days 1 to 14) or chemotherapy alone every 3 weeks for 3 preoperative cycles followed by 5 postoperative cycles. All patients will be followed for survival.

3.2 Study Schema

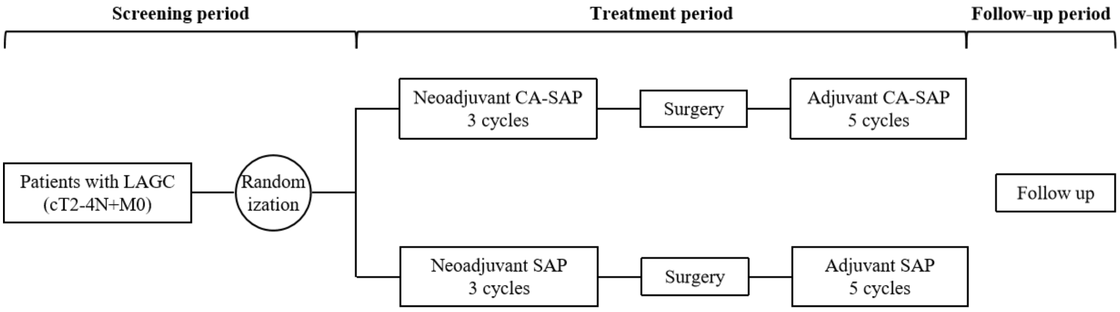

3.3 Study Duration

The total duration for enrollment is expected to be 24 months, beginning with the first patient in June, 2019, and ending with the last patient in June, 2022. The initial analyses for the primary endpoint are planned in the third quarter of 2022. The total duration of the study is expected to be 5 years, including a follow-up period of 3 years. The final analyses for OS and PFS are planned in the third quarter of 2025.

3.4 Sample Size

Based on the assumption of MPR rates of 15% in the SAP group and 35% in the CA-SAP group, a sample size of 53 patients per group is required to detect improvement with 80% power and a one-sided alpha level of 0.1 (Fisher's exact test), including a 5% dropout rate.

4. Study Population

4.1 Inclusion Criteria

- Age older than 18 and younger than 75 years
- Primary gastric adenocarcinoma confirmed pathologically by endoscopic biopsy
- Clinical stage T2-4aN+M0 disease as assessed by CT/MRI, PET-CT, and laparoscopy, if feasible
- At least one measurable lesion according to the RECIST, version 1.1
- ECOG performance status of 0 or 1
- Life expectancy of at least 12 weeks
- Acceptable bone marrow, hepatic, and renal function, including:

blood routine examination (No blood transfusion within 14 days)

White blood cell count >3.0 × 10^9 cells per L and platelet count >100 × 10^9 cells per L

Total bilirubin <1.5 × ULN, alanine aminotransferase or aspartate amino transferase <2.5 × ULN

Serum creatinine ≤1 × ULN, or calculated glomerular filtration rate >60 mL/min

- Written informed consent

4.2 Exclusion Criteria

- Prior chemotherapy, radiotherapy, targeted therapy, or immunotherapy
- Contraindications for surgical treatment or chemotherapy
- Presence of distant metastasis
- History of other malignant disease within the past 5 years, except: basal cell carcinoma of the skin, squamous cell carcinoma of the skin, or in situ cervical cancer
- Any active or history of autoimmune disease, or history of syndrome that required

systemic steroids or immunosuppressive medications, except for subjects with vitiligo or resolved childhood asthma/atopy

- History of immunodeficiency diseases, including human immunodeficiency virus (HIV), or other acquired or congenital immune-deficient disease, or transplantation
- Severe heart, lung, liver, or renal dysfunction
- Severe mental disorder
- Presence of digestive tract obstruction, jaundice, or acute infectious diseases
- Pregnant or breast-feeding women
- Uncontrolled blood pressure on medication (>140/90 mmHg)
- Evidence of bleeding tendency or receiving thrombolytics or anticoagulants
- History of interstitial lung disease or non-infectious pneumonia
- Positive urinary protein (urinary protein ≥2+ or 24h urine protein >1 g)
- Sensitivity to study drugs

4.3 Drop Out/Removal Criteria

- Use of NMPA-approved modern traditional Chinese medicine agents for the treatment of gastric cancer
- Concurrent radiotherapy or other local treatments during the study periods
- Medication, dosing, and treatment methods do not follow the study protocol

4.4 Discontinue Criteria

- Withdrawn of the informed consent
- PD
- Unbearable toxicity after dose reductions
- Pregnancy
- Loss to follow up
- Investigator’s decision that stopping treatment is in the best interest of the patient
- Death

5. Study Treatments

5.1 Handling and Storage

Study drugs must be stored in a secure area under the appropriate physical conditions. Access to and administration of the study drugs will be limited to the investigators and authorized site staff. The investigators must complete storage, handling, dispensing, and infusion information for the study drugs in time.

5.2 Treatment

The study drugs that are used in this trial are outlined below in **Table 1**.

Table 1. Study drugs.

**Product name**	Camrelizumab	Apatinib	Nap-paclitaxel	S-1
**Dosage form**	Lyophilized powder	Tablet	Lyophilized powder	Capsule
**Unit dose strength**	200 mg	250 mg	100 mg	20 mg
**Route of administration**	IV infusion	Oral	IV infusion	Oral
**Storage requirements**	2-8°C. Protect from light and freezing.	<25°C. Protect from light.	20-30°C. Protect from light.	<25°C. Protect from light.

Treatment will be assigned to eligible patients after signed consent forms:

- Camrelizumab is to be administered initially at 200 mg as a 30-minute IV infusion every 3 weeks for 3 preoperative cycles followed by 5 postoperative cycles.
- Apatinib is to be administered initially at 250 mg orally once daily on days 1 to 21 every 3 weeks for 3 preoperative cycles followed by 5 postoperative cycles.
- Nap-paclitaxel is to be administered initially at 125 mg/m^2 as a 30-minute IV infusion every 3 weeks for 3 preoperative cycles followed by 5 postoperative cycles.
- S-1 is to be administered initially at 40-60 mg orally twice daily on days 1 to 14 every 3 weeks for 3 preoperative cycles followed by 5 postoperative cycles.

Table 2. Dose of S-1 depending on BSA.

**BSA**	**Dose**
< 1.25m^2	40mg× 2/day

$1.25m^2$ - $1.5m^2$	50mg× 2/day
> $1.5m^2$	60mg× 2/day

5.3 Dose Modification

Dose modification will be performed based on the severity of toxicities according to the CTCAE, version 5.0. Reasons for dose interruption, delay, or reduction, the supportive measures, and the outcomes will be documented. Dose interruptions are permitted for reasons apart from toxicities, such as medical/surgical events or logistical reasons (e.g., unrelated medical events or elective surgery).

For participants treated with CA-SAP, if a toxicity is considered to be due solely to one investigational agent (i.e., camrelizumab, apatinib, or chemotherapeutic agents), the dose of that agent should be interrupted or delayed in accordance with the guidelines below and the treatment with other agents can continue as scheduled at the discretion of the investigator. When the attribution of toxicity is uncertain, interruption of apatinib and chemotherapeutic agents is done first. If one agent is discontinued due to unacceptable toxicity, patients are able to continue the study with the other agent.

5.3.1 Dose Modification for Camrelizumab

Toxicities associated or possibly associated with camrelizumab may represent an immunologic etiology. For suspected immune-related AEs (irAEs), ensure adequate evaluation to confirm etiology or exclude other causes.

In general, administration of camrelizumab may be continued in the presence of most grade 1 irAEs. For grade 2-4 irAEs, camrelizumab is usually discontinued and can be resumed once irAEs resolve to ≤grade 1. Camrelizumab should be permanently discontinued if the irAE cannot resolve within 12 weeks of the last dose or corticosteroids cannot be reduced to ≤10 mg/day prednisone or equivalent within 12 weeks. Permanent discontinuation of camrelizumab should be considered for any severe or life-threatening events.

The use of corticosteroids is the mainstay of management of irAEs. For severe and

life-threatening irAEs, corticosteroids should be initiated intravenously first followed by oral administration. With improvement to ≤grade 1, initiate corticosteroid taper and continue to taper over at least 1 month. Other immunosuppressive treatment should be initiated if irAEs cannot be controlled by corticosteroids within 3-5 days.

Dose reduction is not permitted for camrelizumab.

- **Immune-mediated Pneumonitis**

Camrelizumab can cause immune-mediated pneumonitis, defined as requiring use of corticosteroids and no clear alternate etiology.

Patients should be monitored for signs with radiographic imaging and for symptoms of pneumonitis. Administer corticosteroids at a dose of 1 mg/kg/day prednisone equivalents for grade 2 pneumonitis. Administer corticosteroids at a dose of 2-4 mg/kg/day prednisone equivalents followed by corticosteroid taper for grade 3-4 pneumonitis. Withhold camrelizumab for grade 2 pneumonitis until resolution for ≤grade 1 pneumonitis. Permanently discontinue camrelizumab for grade 3-4 pneumonitis.

- **Immune-mediated Hepatitis**

Camrelizumab can cause immune-mediated hepatitis, defined as requiring use of corticosteroids and no clear alternate etiology.

Patients should be monitored for abnormal liver tests prior to and periodically during treatment. Administer corticosteroids at a dose of 0.5-1 mg/kg/day prednisone equivalents for grade 2 transaminase elevations. Administer corticosteroids at a dose of 1-2 mg/kg/day prednisone equivalents followed by corticosteroid taper for grade 3-4 transaminase elevations, with or without concomitant elevation in total bilirubin. Withhold camrelizumab for grade 2-3 pneumonitis until resolution for ≤grade 1 pneumonitis. Permanently discontinue camrelizumab for grade 4 or for recurrent transaminase elevations upon re-initiation of camrelizumab.

- **Immune-mediated Colitis**

Camrelizumab can cause immune-mediated colitis, defined as requiring use of corticosteroids with no clear alternate etiology.

Patients should be monitored for signs and symptoms of colitis. Administer corticosteroids at a dose of 0.5-1 mg/kg/day prednisone equivalents followed by corticosteroid taper for grade 2 colitis of more than 5 days duration; if worsening or no improvement occurs despite initiation of corticosteroids, increase dose to 1-2 mg/kg/day prednisone equivalents. Administer corticosteroids at a dose of 1-2

mg/kg/day prednisone equivalents followed by corticosteroid taper for grade 3-4 colitis. In cases of corticosteroid-refractory colitis, consider repeating infectious workup to exclude alternative etiologies. Addition of an alternative immunosuppressive agent to the corticosteroid therapy, or replacement of the corticosteroid therapy should be considered in corticosteroid-refractory immune-mediated colitis if other causes are excluded. Withhold camrelizumab for grade 2-3 colitis until resolution for ≤grade 1 colitis. Permanently discontinue camrelizumab for grade 4 or for recurrent colitis upon re-initiation of camrelizumab.

- **Immune-mediated Endocrinopathies**

Hypothyroidism and Hyperthyroidism

Camrelizumab can cause autoimmune thyroid disorders.

Monitor thyroid function prior to and periodically during camrelizumab treatment. Administer hormone-replacement therapy for hypothyroidism. Initiate medical management for control of hyperthyroidism. Withhold camrelizumab for grade 3 hypothyroidism until initiation of hormone-replacement therapy. Withhold camrelizumab for grade 3 hyperthyroidism until resolution for ≤grade 2 hyperthyroidism. Permanently discontinue camrelizumab for grade 4 hypothyroidism or hyperthyroidism.

Others

Other endocrinopathies include hypophysitis, adrenal insufficiency, type 1 diabetes mellitus, etc. Monitor patients for signs and symptoms. Withhold camrelizumab for grade 2-3. Permanently discontinue camrelizumab for grade 4 endocrinopathies.

- **Immune-Mediated Nephritis and Renal Dysfunction**

Camrelizumab can cause immune-mediated nephritis, defined as renal dysfunction or ≥ grade 2 increased creatinine, requirement for corticosteroids, and no clear alternate etiology.

Monitor patients for elevated serum creatinine prior to and periodically during treatment. Administer corticosteroids at a dose of 0.5-1 mg/kg/day prednisone equivalents for grade 2-3 increased serum creatinine, if worsening or no improvement occurs, increase dose of corticosteroids to 1-2 mg/kg/day prednisone equivalents. Administer corticosteroids at a dose of 1-2 mg/kg/day prednisone equivalents followed by corticosteroid taper for grade 4 increased serum creatinine. Withhold camrelizumab for moderate grade 2 increased serum creatinine until resolution for ≤grade 1 increased serum creatinine. Permanently discontinue camrelizumab for grade 3-4 increased serum

creatinine.

- **Immune-mediated Skin Adverse Reactions**

Camrelizumab can cause immune-mediated rash, including reactive cutaneous capillary endothelial proliferation (RCCEP).

Patients should be monitored for signs and symptoms. Administer corticosteroids at a dose of 1 to 2 mg/kg/day prednisone equivalents followed by a corticosteroid taper for grade 3-4 rash. Withhold camrelizumab for grade 3 rash until resolution for ≤grade 2 rash and permanently discontinue camrelizumab for grade 4 rash.

- **Other Immune-mediated Adverse Reactions**

Camrelizumab can cause other clinically significant and potentially fatal immune-mediated adverse reactions. Immune-mediated adverse reactions may occur after discontinuation of camrelizumab therapy. For any suspected immune-mediated adverse reactions, exclude other causes. Based on the severity of the adverse reaction, permanently discontinue or withhold camrelizumab, administer high-dose corticosteroids, and if appropriate, initiate hormone replacement therapy. Upon improvement to ≤grade 1, initiate corticosteroid taper and continue to taper over at least 1 month. Consider restarting nivolumab after completion of corticosteroid taper based on the severity of the event. Permanently discontinue camrelizumab for severe or life-threatening adverse reactions.

- **Infusion Reactions**

Camrelizumab can cause severe infusion reactions. Discontinue camrelizumab in patients with grade 3-4 infusion reactions. Interrupt or slow the rate of infusion in patients with grade 1-2 infusion reactions.

5.3.2 Dose Modification for Apatinib

Management of treatment-related AEs may require interruption and/or dose reduction of apatinib. The dose reduction levels for apatinib in presence of AEs are listed in **Table 3**. Dose modification of apatinib guidelines are provided in **Table 4**. The dose modifications must be performed as clinically indicated at the discretion of the investigator.

For intolerable grade 2 AEs, apatinib should be interrupted until resolution to ≤grade 1. For grade 3-4 AEs, apatinib should be interrupted until resolution to ≤grade 1 or 2, then resume apatinib with one dose or two dose reductions, or permanently

discontinue treatment which is based on investigator judgement. A maximum of two dose reductions are permitted for apatinib. Permanently discontinue apatinib in the presence of grade 3 or 4 AEs after two dose reductions. However, exception will be permitted in the best interest of the patients. Dose escalation is not permitted.

Table 3. Dose-adjustment criteria for apatinib.

Level	Dose of Apatinib
Starting dose	250 mg once daily
Decrease 1 dose level	250 mg once daily with two days on and one day off
Decrease 2 dose level	250 mg once daily every other day

Table 4. Dose modification guidelines for apatinib.

Toxicity	Grade	Timing for Resumption	Dose Modification for Apatinib
Hematological toxicity	Grade 3	Resolution for ≤grade 2 toxicity	1st occurrence: maintain the same dose.
			2nd occurrence: resume at the next lower dose.
			3rd occurrence: resume at the next lower dose.
	Grade 4	Resolution for ≤grade 2 toxicity	1st occurrence: resume at the next lower dose.
			2nd occurrence: resume at the next lower dose.
Hypertension	Grade 3	Resolution for ≤grade 2 toxicity	1st occurrence: resume at the next lower dose.
			2nd occurrence: resume at the next lower dose.
	Grade 4	Permanently discontinue	Permanently discontinue
Proteinuria	Grade 3	Resolution for ≤grade 2 toxicity	1st occurrence: resume at the next lower dose.
			2nd occurrence: resume at the next lower dose.
Palmar-plantar erythrodysesthesia syndrome	Grade 2 (cannot resolve to ≤grade 1 despite supportive care measures)	Resolution for ≤grade 1 toxicity	1st occurrence: maintain the same dose.
			2nd occurrence: resume at the next lower dose.
			3rd occurrence: resume at the next lower dose.
	Grade 3	Resolution for ≤grade 1	1st occurrence: resume at the next lower dose.

		toxicity	2nd occurrence: resume at the next lower dose.
Fatigue	Grade 2 (cannot resolve to ≤grade 1 despite supportive care measures)	Resolution for ≤grade 1 toxicity	1st occurrence: maintain the same dose.
			2nd occurrence: resume at the next lower dose.
			3rd occurrence: resume at the next lower dose.
	Grade 3	Resolution for ≤grade 1 toxicity	1st occurrence: resume at the next lower dose.
			2nd occurrence: resume at the next lower dose.
Abdominal pain	Grade 2 (cannot resolve to ≤grade 1 despite supportive care measures)	Resolution for ≤grade 1 toxicity	1st occurrence: resume at the next lower dose. 2rd occurrence: resume at the next lower dose.
	Grade 3	Resolution for ≤grade 1 toxicity	
Hemorrhage	Any grade cerebral hemorrhage, grade 2 pulmonary hemorrhage, or grade 3 hemorrhage at any site	Permanently discontinue	Permanently discontinue
Arterial thromboembolism	Any grade	Permanently discontinue	Permanently discontinue
Pulmonary fibrosis	Any grade	Permanently discontinue	Permanently discontinue
Other non-hematological toxicities	Grade 3 or 4	Resolution for ≤grade 1 toxicity	1st occurrence: resume at the next lower dose.
			2nd occurrence: resume at the next lower dose.

5.3.3 Dose Modification for Chemotherapeutic Agents

For intolerable grade 2 AEs, chemotherapeutic agents (nap-paclitaxel and S-1) should be interrupted until resolution to ≤grade 1. For grade 3-4 AEs, chemotherapeutic agents

should be interrupted until resolution to ≤grade 1 or 2, then resume treatment with one dose or two dose reductions, or permanently discontinue treatment which is based on investigator judgement. A maximum of two dose reductions are permitted for chemotherapeutic agents. Permanently discontinue chemotherapeutic agents in the presence of grade 3 or 4 AEs after two dose reductions. However, exception will be permitted in the best interest of the patients. Dose escalation is not permitted. Dose modification of chemotherapeutic agents guidelines are provided in **Table 5**. The dose modifications must be performed as clinically indicated at the discretion of the investigator.

Table 5. Dose modification guidelines for chemotherapeutic agents.

**Toxicity**	**Grade**	**Timing for Resumption**	**Dose Modification for Chemotherapeutic Agents**
Hematological toxicity	Grade 3-4	Resolution for ≤grade 1 toxicity	1st occurrence:<br>75% of Nap-paclitaxel starting dose<br>75% of S-1 starting dose
			2nd occurrence:<br>50% of Nap-paclitaxel starting dose<br>50% of S-1 starting dose
Mucositis/stomatitis, diarrhea, and anorexia	Grade 2	Resolution for ≤grade 1 toxicity	1st occurrence:<br>maintain the same dose
			2nd occurrence:<br>75% of S-1 starting dose
			3rd occurrence:<br>50% of S-1 starting dose
	Grade 3	Resolution for ≤grade 1 toxicity	1st occurrence:<br>75% of S-1 starting dose
			2nd occurrence:<br>50% of S-1 starting dose
	Grade 4	Resolution for ≤grade 1 toxicity	1st occurrence:<br>50% of S-1 starting dose or permanently discontinue
Peripheral neuropathy	Grade 2 (cannot resolve to ≤grade 1 despite supportive care measures)	Resolution for ≤grade 1 toxicity	1st occurrence:<br>75% of Nap-paclitaxel starting dose<br>2nd occurrence:<br>50% of Nap-paclitaxel starting dose
	Grade 3-4	Resolution for ≤grade 1 toxicity	
Other non-hematological toxicity	Grade 2	Resolution for ≤grade 1	1st occurrence:<br>maintain the same dose

		toxicity	2nd occurrence:<br>75% of Nap-paclitaxel starting dose<br>75% of S-1 starting dose
			3rd occurrence:<br>50% of Nap-paclitaxel starting dose<br>50% of S-1 starting dose
	Grade 3	Resolution for ≤grade 1 toxicity	1st occurrence:<br>75% of Nap-paclitaxel starting dose<br>75% of S-1 starting dose
			2nd occurrence:<br>50% of Nap-paclitaxel starting dose<br>50% of S-1 starting dose
	Grade 4	Resolution for ≤grade 1 toxicity	1st occurrence:<br>50% of Nap-paclitaxel and S-1 starting dose or permanently discontinue

5.4 Concomitant Therapy

Concomitant therapy refers to any type of treatment continued from 14 days before randomization, during the study administration period, and until 30 days after the last dose, or all treatment initiated since the first dose of study drugs. Such treatment should be carefully and accurately documented in the Case Report Forms (CRFs).

5.5 Prudently or Forbidden Used Drug

Camrelizumab is a human monoclonal antibody that is not metabolized by cytochrome P450 enzymes or other drug metabolizing enzymes. These are not expected to have pharmacokinetic-based interactions.

Apatinib has a strong inhibition activity on CYP3A4, YP2C9 and CYP2C19 (IC50 <0.5 μM). The CYP3A4 inductor (Dexamethasone, catarrh imipramine, Rifampin, and Phenobarbital), inhibitors (Ketoconazole, Itraconazole, Erythromycin, and Klaricid) and substrates (Simvastatin, Cyclosporine and Pimozide) should be used with caution. Other drugs metabolized by CYP3A4 (such as Benzodiazepines, Dihydropyridine, calcium channel blockers, and HMG-COA reductase inhibitors) and CYP2C9, CYP2C19 substrates should also be used with caution.

6. Study Procedure

6.1 Screening Period

The following items are completed within 2 weeks before randomization:

- Demographics data: age, gender, date of birth, height, weight, etc.
- Tumor data: date of diagnosis, histological classification, clinical stage, etc.
- Medical history: comorbidities, previous medication/surgery, etc.
- Imaging examination: chest, abdomen and pelvis CT or MRI.

After admission, patients who meet the eligibility criteria are screened out. For these patients, the investigator introduces this trial in detail, and the patients sign informed consent if they agree to participate.

The following items are completed within 1 week before randomization:

- ECOG performance status.
- Vital signs: heart rate, breathing rate, temperature, and blood pressure.
- Physical examination: head, skin, lymph nodes, eyes, ears, nose, throat, mouth, respiratory system, cardiovascular system, abdomen, genitourinary system, musculoskeletal system, and nervous system.
- Blood routine: hemoglobin, hematocrit, RBC, WBC, ANC, and platelet count.
- Urinary routine: a 24-hour urinary protein quantification test should be performed in the presence of urine protein ≥2+.
- Stool routine: fecal occult blood.
- Biochemistry: bilirubin, ALT, AST, ALP, creatinine, glucose, calcemia, sodium, and potassium.
- Coagulation test: PT, APTT, TT, and fibrinogen.
- Thyroid function test: TSH, fT3, and fT4.
- Tumor markers: CEA and CA 19-9.
- HBV, HCV, and HIV tests.
- Pretreatment tumor samples.
- 12-lead ECG.
- Pregnancy tests (for women at childbearing age).

6.2 Treatment Period

6.2.1 Neoadjuvant Treatment Period

Administration of the neoadjuvant treatment is initiated within 7 days after randomization. The following items are completed during the neoadjuvant treatment:

- Physical examination, vital signs, ECOG performance status, tumor markers, and ECG are performed on day 1 of cycles 2-3. If cardiac pains or palpitations occur, myocardial enzyme spectrum (creatine kinase, lactate dehydrogenase), ECG, and cardiac ultrasound should be immediately performed.
- Blood routine and biochemistry are performed on day 8 of cycle 1 and days 1, 8 of cycles 2-3.
- Urinary routine, stool routine, coagulation test, thyroid function test, and tumor markers are performed on day 1 of cycles 2-3.
- Imaging examination is performed after completion of cycle 2.
- AEs during the treatment period should be recorded and graded according to the CTCAE, version 5.0.
- Blood pressure monitoring is performed by the patient and recorded in a diary card.

6.2.2 Preoperative Assessments and Surgery

After completion of the last cycle of neoadjuvant treatment, blood routine, biochemistry, stool routine, urine routine, 12-lead ECG, coagulation test, thyroid function test, and tumor markers are performed. Without clear surgical contraindications and with the patients' consent, surgery will be performed 2-4 weeks later. If the disease progresses, laparoscopic exploration is recommended. The following items are completed after surgery:

- Surgical outcomes: operation time, blood loss, intraoperative complications (e.g. major bleeding), postoperative complications and management, postoperative hospital stay, etc.
- Tumor data: tumor regression grade, residual tumor (R) classification, etc.
- Surgical specimens.

6.2.3 Adjuvant Treatment Period

Adjuvant treatment started 3-8 weeks after gastrectomy. The following items are completed during the adjuvant treatment:

- Physical examination, vital signs, ECOG performance status, tumor markers, and

ECG are performed on day 1 of cycles 2-5. If cardiac pains or palpitations occur, myocardial enzyme spectrum (creatine kinase, lactate dehydrogenase), ECG, and cardiac ultrasound should be immediately performed.

- Blood routine and biochemistry are performed on day 8 of cycle 1 and days 1, 8 of cycles 2-5.
- Urinary routine, stool routine, coagulation test, thyroid function test, and tumor markers are performed on day 1 of cycles 2-5.
- Imaging examination is performed every 3 months.
- AEs during the treatment period should be recorded and graded according to the CTCAE, version 5.0.
- Blood pressure monitoring is performed by the patient and recorded in a diary card.

6.3 Follow-up Period

After completion of the last administration of study drugs, patients enter follow-up period. All patients are followed up with every 3 months (± 2 weeks) during the first 2 years and then every 6 months (± 2 weeks) beyond the third year. Data on survival status and disease progression are recorded in the follow-up tables.

Assessments during the follow-up period include physical examination, blood routine, biochemistry, tumor markers, abdominal ultrasound, and chest, abdomen and pelvis CT. Gastroscopy is recommended to performed once a year.

6.4 Progression of Disease

If any of the following criteria is met, it is defined as the progressive disease:

- Determination as PD according to the RECIST, version 1.1 during the neoadjuvant treatment
- Reporting of a distant metastasis from pathology
- R1 or R2 resection
- Finding of a recurrence/distant metastasis during follow-up after R0 resection

For a subject determined to have disease progression, administration of the study drugs will be discontinued and other anti-tumor treatment will be administered at the discretion of the investigator.

7. Efficacy Assessments

- **Pathological Response**

Tumor regression grade (TRG) is evaluated centrally using the Becker regression criteria, which are based on the percentage of vital tumor cells in the tumorous area and include the following categories: TRG 1a (no residual tumor cells), TRG 1b (<10% residual tumor cells), TRG 2 (10-50% residual tumor cells) and TRG 3 (>50% residual tumor cells). The MPR rate is defined as the proportion of patients with <10% residual tumor cells in resection specimens (TRG 1a/1b). More details are provided in **Appendix 1**.

- **Radiologic Response**

Radiologic response is evaluated using RECIST (version 1.1), which is based on CT or MRI findings and includes complete response (CR), partial response (PR), stable disease (SD), and progressive disease (PD). The objective response rate (ORR) was defined as the proportion of patients with CR and PR, and the disease control rate (DCR) was defined as the proportion of patients with CR, PR, and SD. More details are provided in **Appendix 4**.

In a comparison between the pretreatment and posttreatment clinical staging, T downstaging, N downstaging are compared between the two groups based on the CT findings.

- **Surgical Margin**

Tumor condition is explained according to the residual tumor (R) classification:

- R0: No residual cancer (negative cross-section)
- R1: Microscopically observed residual cancer (positive cross-section)
- R2: Macroscopically observed residual cancer

R0 resection rate is defined as the proportion of patients who achieve the R0 resection.

- **Survival**

OS is defined as the time between randomization and the death date. Patients alive at last report will be considered censored at the endpoint. Alive patients will be censored at the last date known to be alive, either during study treatment period or during follow-up period.

PFS is defined as the time between disease progression (see **Section 6.4**) or death irrespective of cause and censored at the date of last contact.

8. Safety Assessments

8.1 Safety Endpoints

Data on all AEs, whether volunteered by the subject, discovered by investigator questioning, or detected through physical examination, laboratory test or other means, will be collected, recorded, and followed as appropriate.

The investigator will collect information on the following variables and record in the CRFs:

- **Surgical safety:** operation time, blood loss, intraoperative complications, postoperative morbidity, mortality, postoperative hospital stay, time to ambulation, time to first flatus, and time to first liquid intake.

- Postoperative morbidity is evaluated according to the Clavien-Dindo classification (**Appendix 6**)

- **Study drug safety:** AEs and serious AEs (SAEs)

- All AEs should be recorded by it duration, regulatory seriousness criteria, suspected relationship to the study drug, and actions taken. AEs are evaluated according to the CTCAE, version 5.0.

- **Details on drug modification caused by AEs**

8.2 Definition of Adverse Event and Serious Adverse Event

An AE is defined as any new untoward medical occurrence or worsening of a preexisting medical condition in a clinical investigation participant administered study drug and that does not necessarily have a causal relationship with this treatment. An AE can therefore be any unfavorable and unintended sign (such as an abnormal laboratory finding), symptom, or disease temporally associated with the use of study drugs, whether or not considered related to the study drugs. The causal relationship to study drug is determined by a physician and should be used to assess all AEs.

The **casual relationship** can be one of the following:

- Definitely: An AE which has a timely relationship to the administration of the study drugs, follows a known pattern of response, for which no alternative cause is present.

- Probably: An AE, which has a timely relationship to the administration of the study drugs, follows a known pattern of response, but for which a potential alternative cause may be present.

- Unlikely: An AE which does not have a timely relationship to the administration of the study drugs, follows no known pattern of response, does not reappear or worsen after re-administration of the study drugs (if applicable), and for which there is evidence that it is related to a cause other than the study drugs.

- Unrelated: An AE, for which there is evidence that it is definitely related to a cause other than the study drugs. In general, there is no timely relationship to the administration of the study drugs, or if there is a timely relationship, the event does not follow a known pattern of response, and there is an alternative cause.

A **SAE** is an AE not classified as serious that, at any time, fulfills one or more of the following criteria:

- Results in death
- Is life threatening
- Requires in-patient hospitalization or prolongation of existing hospitalization
- Results in persistent or significant disability/incapacity
- Results in congenital anomaly/birth defect
- Any important medical event that may not be immediately life threatening or result in death or hospitalization but, based upon appropriate medical judgment, may endanger the patient or may require intervention to prevent one of the other outcomes listed in the definition above.
- Suspected transmission of an infectious agent (e.g., pathogenic or nonpathogenic) via the study drug is an SAE.
- Any component of a study endpoint that is considered related to study therapy should be reported as an SAE (e.g. death is an endpoint, if death occurred due to anaphylaxis, anaphylaxis must be reported).

Events initially reported as an AE may also become serious.

8.3 Recording and Follow-up of (Serious) Adverse Events

AEs and SAEs will be recorded from time of signature of informed consent, throughout the treatment period and including 30 days after the last dose of study drugs (SAEs will be collected for 90 days after discontinuation of treatment). During the course of the

study all AEs and SAEs should be followed up for each patient. Every effort should be made to obtain a resolution for all events, even if the events continue after discontinuation or study completion.

The investigator is requested to assess the relationship between the study drugs and the occurrence of each (S)AE. The investigator will use clinical judgment to determine the relationship. Alternatives causes, such as natural history of the underlying diseases, concomitant therapy, other risk factors, and the temporal relationship of the event to the investigational product will be considered and investigated.

All SAEs occurring to any patient on this study, regardless of attribution, should be reported to the Institutional Review Board and regulatory authority within 24 hours of the investigator's knowledge of the events. The report of SAE should be consistent with standard SAE reporting guidelines.

9. Statistical Analysis Plan

9.1 Sample Size Considerations

The primary endpoint of this study is the MPR rate. Based on the assumption of MPR rates of 15% in the SAP group and 35% in the CA-SAP group, a sample size of 53 patients per group is required to detect improvement with 80% power and a one-sided alpha level of 0.1 (Fisher's exact test), including a 5% dropout rate.

9.2 Analysis Sets

- Intention-to-treat (ITT) set included all patients who are randomly assigned. This population will be used for the efficacy analyses.
- Per-protocol set included patients in the ITT population who did not present major deviations from the protocol. We pre-specified that participants who received surgery after administration of neoadjuvant CA-SAP or SAP are included in this set. This population will be also used for the efficacy analyses.
- Safety analysis set included patients who received at least one dose of allocated treatment. This population will be also used for the safety analyses.

9.3 Statistical Analysis

Baseline characteristics and demographic variables will be summarized in the ITT set. Surgical and pathology results will be summarized in the per-protocol set. Nonsurgical AEs will be summarized in the safety analysis set. Continuous variables are presented as medians and interquartile ranges (IQRs) or means and standard deviations (SDs) and are compared using the Wilcoxon rank sum test or t test. Categorical variables are presented as frequencies and percentages and are compared using the $\chi 2$ test or Fisher's exact test.

The efficacy analysis will be performed using both the ITT and per-protocol sets. The efficacy endpoints, including the MPR rate, R0 resection rate, ORR, DCR, and survival, are compared using the Fisher's exact test. The MPR rate, R0 resection rate, ORR, and DCR will be summarized (i.e., number of patients [%]) and 95% confidence intervals (CIs) for the objective response rate will be provided. Medians and 95% confidence intervals (CIs) for OS and PFS will be calculated. Kaplan-Meier plots of OS and PFS will be presented. Hazard ratio assessment and the corresponding 95% CIs can be presented using the Cox proportional hazard model.

The safety analysis will be done on the safety analysis set. All AEs will be presented as frequencies and percentages in each category. If a patient experiences the same AE multiple times during the treatments, the event will be counted only once and by the greatest severity.

10. Data Management

10.1 CRF Completion Guidelines

For each participant, CRF should be completed by the investigator. The investigator is responsible for ensuring that data recorded in the CRFs are complete, accurate, and legible. The CRF will be served as the original records, and should not be changed. Any correction must not be made on the original records, but an additional narrative can be used for recording the change, the investigator should sign and date for any change made. All data in the CRFs must come from and be consistent with the source documents, i.e. patient's file or medical records.

The CRFs should be completed before reviewing by the Monitor. For any questionable data in the case report form, the Monitor will issue the Data Request (DRQ) to query the investigator, the investigator should answer it as soon as possible and return.

10.2 Data Lock

After validating the data correction under blinding review, the principle investigator and statistical analysis personnel will lock the data. The locked database is no longer to be changed.

10.3 Quality Control and Quality Assurance

- Investigational personnel must be physicians through training of clinical study and who will work under the guidance of the principal investigator.
- Prior to the study initiation, the pre-study qualification is needed to ensure the facility is equipped with all rescue equipment and meets the standard requirements.
- Recommendations to study nurses who will administer study medications to subjects, have a good understanding of the drug administration, and ensuring the compliance of study subjects.
- Each study center must strictly conduct in accordance with the study protocol, and complete CRFs faithfully.
- The Monitor will monitor the clinical study according to the Good Clinical Practice and the Standard Operating Procedure, ensure the data recording and reporting is correct and intact, all CRFs will be completed correctly and be verified with the original documents, and ensure the study can be conducted according to the study protocol.

11. Ethics and Regulatory Considerations

11.1 Local Regulations / Helsinki Declaration

The study was performed in accordance with the Helsinki Declaration of 1964 and later

versions, Guidelines for Good Clinical Practice, and Chinese law.

11.2 Informed Consent

The investigator must explain to subjects that their participation is voluntary and that they may withdraw at any study stage at any time, and will not affect their medical treatment and interest; they also can continue to receive other kind of treatments after withdrawal. Subjects must be informed that their participation and their personal information in the study are confidential. Subjects must be informed of the study nature, the study objectives, anticipated benefits, potential hazards or inconveniences, other available treatments, and subject rights and obligations that meet the Declaration of Helsinki, etc. Subjects should be given sufficient time to consider whether or not they will participate the trial. The signature of the participant is needed on the informed consent form. If new safety information changes the risk/benefit assessments, the informed consent may be modified as necessary. If any modification is made, all subjects (including those who have received treatment) will be notified of the new information and given a revised consent form to continue their participation in the study.

11.3 Independent Ethics Committee/Institutional Review Board

Before the study begins, all participating centers should submit the study protocol and relevant documents (CRFs, consent forms, and other documents that may be required) to the Independent Ethics Committee (IEC)/Institutional Review Board (IRB). The study can only start after obtaining approval from the IEC/IRB. Any amendments to the study protocol must be submitted to the IEC/IRB in accordance with local laws and regulations.

11.4 Confidentiality Agreement

Data obtained by this study is confidential, and disclosure to third parties other than the regulatory authority, the principle investigator, and study personnel is prohibited.

11.5 Record Storage

The investigator should arrange for the storage of the research files until the end of the study. All records and documents pertaining to the conduct of this study and the distribution of the study drugs, including CRFs, consent forms, laboratory test results, and medical inventory records, must be retained by the principal investigator for at least 5 years.

11.6 Study Interruption

The principal investigator can decide to discontinue this study at any time and for any reason; the decision to discontinue the study will be communicated in writing to the participating researchers. Likewise, if other participating researchers decide to withdraw from the study, they must notify the company in writing.

11.7 Protocol Modification

Any modification to the protocol will be recorded in a written revision and signed by the principal investigator. A signed, revised version will be attached to the last version of the protocol.

References

[1] Bray F, Ferlay J, Soerjomataram I, et al. Global cancer statistics 2018: GLOBOCAN estimates of incidence and mortality worldwide for 36 cancers in 185 countries. CA Cancer J Clin 2018; 68: 394-424.

[2] Yang L, Zheng R, Wang N, et al. Incidence and mortality of stomach cancer in China,2014. Chin J Cancer Res 2018; 30: 291-298.

[3] Digklia A, Wagner AD. Advanced gastric cancer: Current treatment landscape and future perspectives. World J Gastroenterol 2016; 22: 2403-2414.

[4] Smyth EC, Nilsson M, Grabsch HI, et al. Gastric cancer. Lancet 2020; 396: 635-648.

[5] Wilke H, Preusser P, Fink U, et al.Preoperative chemotherapy in locally advanced and nonresectable gastric cancer: a phase II study with etoposide, doxorubicin, and cisplatin. J Clin Oncol 1989; 7: 1318-1326.

[6] Paoletti X, Oba K, Burzykowski T, et al.Benefit of adjuvant chemotherapy for resectable gastric cancer: a meta-analysis. JAMA 2010; 303: 1729-1737.

[7] Cunningham D, Allum WH, Stenning SP, et al. Perioperative chemotherapy versus surgery alone for resectable gastroesophageal cancer. N Engl J Med 2006; 355: 11-20.

[8] Ychou M, Boige V, Pignon JR, et al. Perioperative chemotherapy compared with surgery alone for resectable gastroesophageal adenocarcinoma: an FNCLCC and FFCD multicenter phase III trial. J Clin Onco 2011; 29: 1715-1721.

[9] Al-Batran SE, Homann N, Pauligk C, et al. Perioperative chemotherapy with fluorouracil plus leucovorin, oxaliplatin, and docetaxel versus fluorouracil or capecitabine plus cisplatin and epirubicin for locally advanced, resectable gastric or gastro-oesophageal junction adenocarcinoma (FLOT4): a randomised, phase 2/3 trial. Lancet 2019; 393: 1948-1957.

[10] Bu Z, Ji J. Comments on Chinese guidelines for diagnosis and treatment of gastric cancer 2018 (English edition). Chin J Cancer Res 2020; 32: 446-447.

[11] Huang D, Ba Y, Xiong J, et al. A multicentre randomised trial comparing weekly paclitaxel + S-1 with weekly paclitaxel + 5-fluorouracil for patients with advanced gastric cancer. Eur J Cancer 2013; 49: 2995-3002.

[12] Ishigami H, Fujiwara Y, Fukushima R, et al. Phase III Trial Comparing Intraperitoneal and Intravenous Paclitaxel Plus S-1 Versus Cisplatin Plus S-1 in Patients With Gastric Cancer With Peritoneal Metastasis: PHOENIXGC Trial. J Clin Oncol

2018; 36: 1922-1929.

[13] Gradishar WJ, Tjulandin S, Davidson N, et al. Phase III trial of nanoparticle albumin-bound paclitaxel compared with polyethylated castor oil-based paclitaxel in women with breast cancer. J Clin Oncol 2005; 23: 7794-7803.

[14] Guan ZZ, Li QL, Feng FY, et al. Superior efficacy of a Cremophor-free albumin-bound paclitaxel compared with solvent-based paclitaxel in Chinese patients with metastatic breast cancer. Asia Pac J Clin Oncol 2009; 5:165-174.

[15] Zhang C, Awasthi N, Schwarz MA, et al. Superior antitumor activity of nanoparticle albumin-bound paclitaxel in experimental gastric cancer. PLoS One 2013; 8: e58037.

[16] Ryu MH, Yoo C, Kim JG, et al. Multicenter phase II study of trastuzumab in combination with capecitabine and oxaliplatin for advanced gastric cancer. Eur J Cancer 2015; 51: 482-488.

[17] Iqbal S, Goldman B, Fenoglio-Preiser CM, et al. Southwest Oncology Group study S0413: a phase II trial of lapatinib (GW572016) as first-line therapy in patients with advanced, or metastatic gastric cancer. Annals of Oncology 2011; 22: 2610.

[18] Sun W, Powell M, O'Dwyer PJ, et al. Phase II study of sorafenib in combination with docetaxel and cisplatin in the treatment of metastatic or advanced gastric and gastroesophageal junction adenocarcinoma: ECOG5203. J Clin Oncol 2010; 28: 2947-2951.

[19] Ohtsu A, Ajani JA, Bai YX, et al. Everolimus for previously treated advanced gastric cancer: results of the randomized, double-blind, phase III GRANITE-1 study. J Clin Oncol 2013; 31: 3935-3943.

[20] Hicklin DJ, Ellis LM. Role of the vascular endothelial growth factor pathway in tumor growth and angiogenesis. J Clin Oncol 2005; 23: 1011-1027.

[21] Tian S, Quan H, Xie C, et al. YN968D1 is a novel and selective inhibitor of vascular endothelial growth factor receptor-2 tyrosine kinase with potent activity in vitro and in vivo. Cancer Sci 2011; 102: 1374-1380.

[22] Li J, Qin SK, Xu JM, et al. Randomized, Double-Blind, Placebo-Controlled Phase III Trial of Apatinib in Patients With Chemotherapy-Refractory Advanced or Metastatic Adenocarcinoma of the Stomach or Gastroesophageal Junction. J Clin Oncol 2016, 34: 1448-1454.

[23] Lin JX, Xu YC, Lin W, et al. Effectiveness and Safety of Apatinib Plus Chemotherapy as Neoadjuvant Treatment for Locally Advanced Gastric Cancer: A

Nonrandomized Controlled Trial. JAMA Netw Open 2021; 4: e2116240.

[24] Zhang X, Schwartz JC, Guo X, et al. Structural and functional analysis of the costimulatory receptor programmed death-1. Immunity 2004; 20: 337-347.

[25] Dong H, Strome SE, Salomao DR, et al. Tumor-associated B7-H1 promotes T-cell apoptosis: a potential mechanism of immune evasion. Nat Med 2002; 8: 793-800.

[26] Thompson RH, Kuntz SM, Leibovich BC, et al. Tumor B7-H1 is associated with poor prognosis in renal cell carcinoma patients with long-term follow-up. Cancer Res 2006; 66: 3381-3385.

[27] Shitara K, Özgüroğlu M, Bang YJ, et al. Pembrolizumab versus paclitaxel for previously treated, advanced gastric or gastro-oesophageal junction cancer (KEYNOTE-061): a randomised, open-label, controlled, phase 3 trial. Lancet 2018; 392: 123-133.

[28] Bang YJ, Kang YK, Catenacci DV, et al. Pembrolizumab alone or in combination with chemotherapy as first-line therapy for patients with advanced gastric or gastroesophageal junction adenocarcinoma: results from the phase II nonrandomized KEYNOTE-059 study. Gastric Cancer 2019; 22: 828-837.

[29] Huang Y, Yuan J, Righi E, et al. Vascular normalizing doses of antiangiogenic treatment reprogram the immunosuppressive tumor microenvironment and enhance immunotherapy. Proc Natl Acad Sci U S A 2012; 109: 17561-17566.

[30] Schmittnaegel M, Rigamonti N, Kadioglu E, et al. Dual angiopoietin-2 and VEGFA inhibition elicits antitumor immunity that is enhanced by PD-1 checkpoint blockade. Sci Transl Med 2017; 9: eaak9670.

[31] Motzer RJ, Atkins MB, Escudier B, et al. IMmotion151: A Randomized Phase III Study of Atezolizumab Plus Bevacizumab vs Sunitinib in Untreated Metastatic Renal Cell Carcinoma (mRCC). J Clin Oncol 2018; 36:6_suppl, 578-578.

[32] Xu J, Zhang Y, Jia R, et al. Anti-PD-1 Antibody SHR-1210 Combined with Apatinib for Advanced Hepatocellular Carcinoma, Gastric, or Esophagogastric Junction Cancer: An Open-label, Dose Escalation and Expansion Study. Clin Cancer Res 2019; 25: 515-523.

Appendix 1 Becker Regression Criteria

Becker Regression Criteria

Grade	Characteristic
TRG 1a	Complete tumor regression without residual tumor
TRG 1b	<10% residual tumor
TRG 2	10% to 50% residual tumor
TRG 3	50% residual tumor cells

Note: The Becker regression criteria are based on the estimation of the percentage of vital tumor cells in relation to the macroscopically identifiable tumor bed.

Appendix 2 Pathological Staging System

American Joint Committee on Cancer (AJCC) TNM Staging Classification for Carcinoma of the Stomach (8th ed, 2017)

- **Definition of Primary Tumor (T)**

T_x Primary tumor cannot be assessed

T_0 No evidence of primary tumor

Tis Carcinoma in situ: intraepithelial tumor without invasion of the lamina propria, high-grade dysplasia

T_1 Tumor invades lamina propria, muscularia mucosa or sub-mucosa

T_{1a} Tumor invades the lamina propria or muscularis mucosae

T_{1b} Tumor invades the sub-mucosa

T_2 Tumor invades the muscularis propria

T_3 Tumor penetrates the subserosal connective tissue without invasion of the visceral peritoneum or adjacent structures

T_4 Tumor invades the serosa (visceral peritoneum) or adjacent structures

T_{4a} Tumor invades the serosa (visceral peritoneum)

T_{4b} Tumor invades adjacent structures/organs

- **Definition of Regional Lymph Node (N)**

N_x Regional lymph node(s) cannot be assessed

N_0 No regional lymph node metastasis

N_1 Metastasis in one or two regional lymph nodes

N_2 Metastasis in three to six regional lymph nodes

N_3 Metastasis in seven or more regional lymph nodes

N_{3a} Metastasis in seven to 15 regional lymph nodes

N_{3b} Metastasis in 16 or more regional lymph nodes

- **Definition of Distant Metastasis (M)**

M_0 No distant metastasis

M_1 Distant metastasis

Post-Neoadjuvant Therapy (ypTNM)

When T is...	And N is...	And M is...	Then the stage group is...
T1	N0	M0	I
T2	N0	M0	I
T1	N1	M0	I
T3	N0	M0	II
T2	N1	M0	II
T1	N2	M0	II
T4a	N0	M0	II
T3	N1	M0	II
T2	N2	M0	II
T1	N3	M0	II
T4a	N1	M0	III
T3	N2	M0	III
T2	N3	M0	III
T4b	N0	M0	III
T4b	N1	M0	III
T4a	N2	M0	III
T3	N3	M0	III
T4b	N2	M0	III
T4b	N3	M0	III
T4a	N3	M0	III
AnyT	AnyN	MI	IV

Appendix 3 Surgical Manual

In this study, the basic purpose of surgery is curative resection. Curative resection is defined as follows:

- No distant metastasis.
- No infiltration to surrounding organs, or in case of infiltration, curative combined resection should be performed.
- No macroscopic residual cancer.
- D2 or greater resection is necessarily required.
- No infiltration of cancer cells on both margins from histopathology.

Selection of gastrectomy The standard surgical procedure for clinically node-positive (cN+) or T2-T4a tumors is either total or distal gastrectomy. Distal gastrectomy is selected when a satisfactory proximal resection margin (see below) can be obtained. When obtaining proximal resection margin is not possible, total gastrectomy is selected.

Resection margin A sufficient resection margin should be ensured when determining the resection line in gastrectomy with curative intent. Proximal margin of at least 3 cm is recommended for T2 or deeper tumors with an expansive growth pattern (types 1 and 2) and 5 cm for those with an infiltrative growth pattern (types 3 and 4). When these rules cannot be satisfied, it is advisable to examine the whole thickness of proximal resection margin by frozen section. For tumors invading the esophagus, resection margin >5 cm is not necessarily required, but frozen section examination of the resection line is preferable to ensure an R0 resection.

Definition of the D levels

- **Total gastrectomy**

D0: Lymphadenectomy less than D1.

D1: No. 1-7.

D1+: D1 + No. 8a, 9, 11p.

D2: D1 + No. 8a, 9, 11p, 11d, 12a.

For tumors invading the esophagus, resection of No.110 should be added to D1+, and resection of Nos. 19, 20, 110 and 111 to D2.

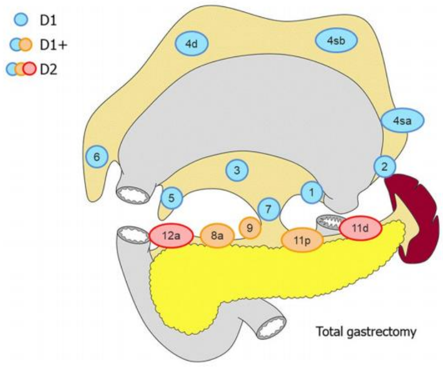

- **Distal gastrectomy**

D0: Lymphadenectomy less than D1.

D1: No. 1, 3, 4sb, 4d, 5, 6, 7.

D1+: D1 + No. 8a, 9.

D2: D1 + No. 8a, 9, 11p, 12a.

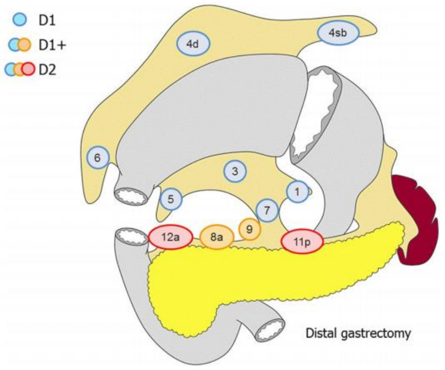

- **Proximal gastrectomy**

D0: Lymphadenectomy less than D1.

D1: No. 1, 2, 3a, 4sa, 4sb, 7.

D1+: D1 + No. 8a, 9, 11p.

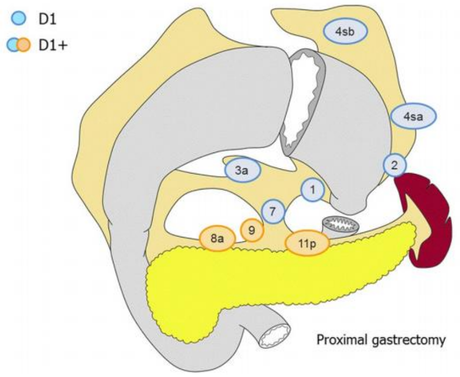

Appendix 4 Response Evaluation Criteria in Solid Tumors (RECIST), Version 1.1

Measurability of tumor at baseline

At baseline, tumor lesions/lymph nodes will be categorised measurable or non-measurable as follows:

- Measurable

Tumor lesions: Must be accurately measured in at least one dimension (longest diameter in the plane of measurement is to be recorded) with a minimum size of:

• 10 mm by CT scan (CT scan slice thickness no greater than 5 mm).

• 10 mm caliper measurement by clinical exam (lesions which cannot be accurately measured with calipers should be recorded as non-measurable).

• 20 mm by chest X-ray.

Malignant lymph nodes: To be considered pathologically enlarged and measurable, a lymph node must be ≥15 mm in short axis when assessed by CT scan (CT scan slice thickness recommended to be no greater than 5 mm). At baseline and in follow-up, only the short axis will be measured and followed.

- Non-measurable

All other lesions, including small lesions (longest diameter <10 mm or pathological lymph nodes with ≥10 to <15 mm short axis) as well as truly non-measurable lesions. Lesions considered truly non-measurable include: leptomeningeal disease, ascites, pleural or pericardial effusion, inflammatory breast disease, lymphangitic involvement of skin or lung, abdominal masses/abdominal organomegaly identified by physical exam that is not measurable by reproducible imaging techniques.

Response criteria

This section provides the definitions of the criteria used to determine objective tumour response for target lesions.

- **Evaluation of target lesions**

Complete Response (CR): Disappearance of all target lesions. Any pathological lymph nodes (whether target or non-target) must have reduction in short axis to <10 mm.

Partial Response (PR): At least a 30% decrease in the sum of diameters of target lesions, taking as reference the baseline sum diameters.

Progressive Disease (PD): At least a 20% increase in the sum of diameters of target lesions, taking as reference the smallest sum on study (this includes the baseline sum if that is the smallest on study). In addition to the relative increase of 20%, the sum must also demonstrate an absolute increase of at least 5 mm. (Note: the appearance of one or more new lesions is also considered progression).

Stable Disease (SD): Neither sufficient shrinkage to qualify for PR nor sufficient increase to qualify for PD, taking as reference the smallest sum diameters while on study。

- **Evaluation of non-target lesions**

This section provides the definitions of the criteria used to determine the tumour response for the group of non-target lesions.

While some non-target lesions may actually be measurable, they need not be measured and instead should be assessed only qualitatively at the time points specified in the protocol.

Complete Response (CR): Disappearance of all non-target lesions and normalisation of tumour marker level. All lymph nodes must be non-pathological in size (<10 mm short axis).

Non-CR/Non-PD: Persistence of one or more non-target lesion(s) and/or maintenance of tumour marker level above the normal limits.

Progressive Disease (PD): Unequivocal progression (see comments below) of existing non-target lesions. (Note: the appearance of one or more new lesions is also considered progression).

Appendix 5 ECOG Performance Status

According to the simplified performance status score scale developed by the ECOG, the patients' performance status can be classified into 6 levels, namely 0-5, as follows:

ECOG Performance Status

Grade	Characteristic
0	Fully active, able to carry on all pre-disease performance without restriction.
1	Restricted in physically strenuous activity but ambulatory and able to carry out work of a light or sedentary nature, e.g., light housework, office work.
2	Ambulatory and capable of all self-care but unable to carry out any work activities. Up and about more than 50% of waking hours.
3	Capable of only limited self-care, confined to bed or chair more than 50% of waking hours.
4	Completely disabled. Cannot carry on any self-care. In total, confined to bed or chair.
5	Dead.

Note: Patients at levels 3, 4 and 5 are generally considered to be unsuitable for surgical treatment or chemotherapy.

Appendix 6 Classification of Surgical Complications

Clavien-Dindo Classification

Grade	Characteristic
**Grade I**	Any deviation from the normal postoperative course without the need for pharmacological treatment or surgical, endoscopic, and radiological interventions. Allowed therapeutic regimens are: drugs as antiemetics, antipyretics, analgetics, diuretics, electrolytes, and physiotherapy. This grade also includes wound infections opened at the bedside.
**Grade II**	Requiring pharmacological treatment with drugs other than such allowed for grade I complications.
**Grade III**	Requiring surgical, endoscopic or radiological intervention<br>**Grade IIIa:** Intervention not under general anesthesia<br>**Grade IIIb:** Intervention under general anesthesia
**Grade IV**	Life-threatening complication requiring ICU management<br>**Grade IVa:** Single organ dysfunction (including dialysis)<br>**Grade IVb:** Multiorgan dysfunction
**Grade V**	Death of a patient.

Efficacy and Safety of Camrelizumab and Apatinib Combined with Nab-paclitaxel and S-1 as Neoadjuvant Treatment for Locally Advanced Gastric Cancer: A Multicenter Randomized Controlled Trial

CLINICAL STUDY PROTOCOL

Registration Number: NCT04195828

Principle Investigator: Chang-Ming Huang, Ph.D.
Department of Gastric Surgery, Fujian Medical University Union Hospital, No. 29 Xinquan Road, Fuzhou, Fujian Province, China.
Telephone: +86-591-83363366
E-mail: hcmlr2002@163.com

Coordinating Investigator: Kai Ye, Ph.D.
Second Affiliated Hospital of Fujian Medical University
E-mail: Yekai1972@126.com

Jian-Chun Cai, Ph.D.
Zhongshan Hospital of Xiamen University
E-mail: jianchunfh2@sina.com

Li-Sheng Cai, Ph.D.
Zhangzhou Municipal Hospital of Fujian Province
E-mail: 1272110762@qq.com

Wei Lin, Ph.D.
The Affiliated Hospital of Putian University
E-mail: linwbj@outlook.com

TABLE OF CONTENTS

Abbreviations 1
SYNOPSIS 3
Study Plan 8
1. Background 10
2. Objectives 13
2.1 Primary Objective 13
2.2 Secondary Objectives 13
2.3 Exploratory Objective 13
3. Study Design and Sample Size 14
3.1 Study Design 14
3.2 Study Schema 14
3.3 Study Duration 14
3.4 Sample Size 14
4. Study Population 15
4.1 Inclusion Criteria 15
4.2 Exclusion Criteria 15
4.3 Drop Out/Removal Criteria 16
4.4 Discontinue Criteria 16
5. Study Treatments 17
5.1 Handling and Storage 17
5.2 Treatment 17
5.3 Dose Modification 18
5.3.1 Dose Modification for Camrelizumab 18
5.3.2 Dose Modification for Apatinib 21
5.3.3 Dose Modification for Chemotherapeutic Agents 23
5.4 Concomitant Therapy 25
5.5 Prudently or Forbidden Used Drug 25
6. Study Procedure 26
6.1 Screening Period 26
6.2 Treatment Period 26
6.2.1 Neoadjuvant Treatment Period 27
6.2.2 Preoperative Assessments and Surgery 27
6.2.3 Adjuvant Treatment Period 27
6.3 Follow-up Period 28
6.4 Progression of Disease 28
7. Efficacy Assessments 29
8. Safety Assessments 30
8.1 Safety Endpoints 30
8.2 Definition of Adverse Event and Serious Adverse Event 30
8.3 Recording and Follow-up of (Serious) Adverse Events 31
9. Statistical Analysis Plan 32
9.1 Sample Size Considerations 32

9.2 Analysis Sets 32
9.3 Statistical Analysis 33
10. Data Management 33
10.1 CRF Completion Guidelines 33
10.2 Data Lock 34
10.3 Quality Control and Quality Assurance 34
11. Ethics and Regulatory Considerations 35
11.1 Local Regulations / Helsinki Declaration 35
11.2 Informed Consent 35
11.3 Independent Ethics Committee/Institutional Review Board 35
11.4 Confidentiality Agreement 36
11.5 Record Storage 36
11.6 Study Interruption 36
11.7 Protocol Modification 36
References 37
Appendix 1 Becker Regression Criteria 40
Appendix 2 Pathological Staging System 41
Appendix 3 Surgical Manual 43
Appendix 4 Response Evaluation Criteria in Solid Tumors (RECIST), Version 1.1 45
Appendix 5 ECOG Performance Status 47
Appendix 6 Classification of Surgical Complications 48

Abbreviations

Abbreviations	Full names
AE	Adverse event
ALT	Alanine aminotransferase
ALP	Alkaline phosphatase
ANC	Absolute neutrophil count
APTT	Activated partial thromboplastin time
AST	Aspartate aminotransferase
BSA	Body surface area
BUN	Blood urea nitrogen
CA	Carbohydrate antigen
CEA	Carcinoembryonic antigen
CI	Confidence interval
CR	Complete response
CRF	Case Report Form
CT	Computed tomography
CTCAE	Common Terminology Criteria for Adverse Events
DFS	Disease-free survival
DRQ	Data Request
ECG	Electrocardiography
ECOG	Eastern Cooperative Oncology Group
GC	Gastric cancer
HBV	Hepatitis B virus
HCV	Hepatitis C virus
HIV	Human immunodeficiency virus
IEC	Independent Ethics Committee
IQR	Interquartile range
IRB	Institutional Review Board

**Abbreviations**	**Full names**
irAE	Immune-related adverse event
LAGC	Locally advanced gastric cancer
mITT	Modified intention-to-treat
MPR	Major pathological response
MRI	Magnetic resonance imaging
NMPA	National Medical Products Administration
OB	Occult blood
ORR	Objective response rate
OS	Overall survival
PD	Progression disease
PD-1	Programmed cell death protein 1
PD-L1	Programmed cell death protein-ligand 1
PET	Positron emission tomography
PFS	Progression-free survival
PR	Partial response
PT	Prothrombin time
RBC	Red blood cell
RCCEP	Reactive cutaneous capillary endothelial proliferation
SAE	Serious adverse event
SD	Stable disease
TRG	Tumor regression grade
TSH	Thyroid-stimulating hormone
TT	Thrombin time
RECIST	Response Evaluation Criteria in Solid Tumors
ULN	Upper limits of normal
VEGF	Vascular endothelial growth factor
WBC	White blood cell

SYNOPSIS

**Title**	Efficacy and Safety of Camrelizumab and Apatinib Combined with Nab-paclitaxel and S-1 as Neoadjuvant Treatment for Locally Advanced Gastric Cancer:<br>A Multicenter Randomized Controlled Trial
**Study Subjects**	Patients with locally advanced gastric cancer (LAGC)
**Study Design**	Multicenter, open-label, randomized phase 2 clinical trial
**Study Objectives**	**Primary endpoint**<br>➢ Major pathological response (MPR)<br>**Secondary endpoints**<br>➢ Radiologic response according to the Response Evaluation Criteria in Solid Tumors (RECIST), version 1.1<br>➢ R0 resection rate<br>➢ Safety of study drugs according to the Common Terminology Criteria for Adverse Events (CTCAE), version 5.0<br>➢ Surgical safety: including morbidity, mortality, hospital stay, etc.<br>➢ Overall survival (OS), Progression-free survival (PFS)
**Number of Patients**	53 patients per group
**Inclusion Criteria**	● Age older than 18 and younger than 75 years<br>● Primary gastric adenocarcinoma confirmed pathologically by endoscopic biopsy<br>● Clinical stage T2-4aN+M0 disease as assessed by CT/MRI, PET-CT, and laparoscopy, if feasible<br>● At least one measurable lesion according to the RECIST, version 1.1<br>● Eastern Cooperative Oncology Group (ECOG) performance status of 0 or 1<br>● Life expectancy of at least 12 weeks<br>● Acceptable bone marrow, hepatic, and renal function, including:

	blood routine examination(No blood transfusion within 14 days)<br>1. White blood cell count >3.0 × 10^9 cells per L and platelet count >100 × 10^9 cells per L<br>2. Total bilirubin <1.5 × ULN, alanine aminotransferase or aspartate amino transferase <2.5 × ULN<br>3. Serum creatinine ≤1 × ULN, or calculated glomerular filtration rate >60 mL/min<br>● Written informed consent
**Exclusion Criteria**	● Prior chemotherapy, radiotherapy, targeted therapy, or immunotherapy<br>● Contraindications for surgical treatment or chemotherapy<br>● Presence of distant metastasis<br>● History of other malignant disease within the past 5 years, except: basal cell carcinoma of the skin, squamous cell carcinoma of the skin, or in situ cervical cancer<br>● Any active or history of autoimmune disease, or history of syndrome that required systemic steroids or immunosuppressive medications, except for subjects with vitiligo or resolved childhood asthma/atopy<br>● History of immunodeficiency diseases, including human immunodeficiency virus (HIV), or other acquired or congenital immune-deficient disease, or transplantation<br>● Severe heart, lung, liver, or renal dysfunction<br>● Severe mental disorder<br>● Presence of digestive tract obstruction, jaundice, or acute infectious diseases<br>● Pregnant or breast-feeding women<br>● Uncontrolled blood pressure on medication (>140/90 mmHg)<br>● Evidence of bleeding tendency or receiving thrombolytics or anticoagulants<br>● History of interstitial lung disease or non-infectious pneumonia<br>● Positive urinary protein (urinary protein≥2+ or 24h urine

	protein >1 g)<br>● Sensitivity to study drugs
**Drop out/removal criteria**	● Use of NMPA-approved modern traditional Chinese medicine agents for the treatment of gastric cancer<br>● Concurrent radiotherapy or other local treatments during the study periods<br>● Medication, dosing, and treatment methods do not follow the study protocol
**Discontinue Criteria**	● Withdrawn of the informed consent<br>● Progression disease (PD)<br>● Unbearable toxicity after dose reductions<br>● Pregnancy<br>● Loss to follow up<br>● Investigator's decision that stopping treatment was in the best interest of the patient<br>● Death
**Study Duration**	● Planned start date: June 1, 2020<br>● Planned enrollment completion date: June 1, 2022<br>● Planned study end date: June 1, 2025
**Treatment Regimens**	**Neoadjuvant treatment**<br>**Treatment group (CA-SAP):**<br>● Camrelizumab, 200 mg intravenously on day 1;<br>● Apatinib, 250 mg orally once daily on days 1 to 21;<br>● Nap-paclitaxel, 125 mg/m^2 intravenously on days 2 and 9;<br>● S-1 (Tigio), 40-60 mg orally twice daily on days 1 to 14.<br>**Control group (SAP):**<br>● Nap-paclitaxel, 125 mg/m^2 intravenously on days 1 and 8;<br>● S-1 (Tigio), 40-60 mg orally twice daily on days 1 to 14.<br>The dose of S-1 is based on body surface area (BSA):<br><table><tr><th>BSA</th><th>Dose</th></tr></table>

< 1.25m^2	40mg× 2/day
1.25m^2 - 1.5m^2	50mg× 2/day
> 1.5m^2	60mg× 2/day

The above treatments will be administered every 3 weeks for three preoperative cycles.

Surgery

Surgery was scheduled 2 to 4 weeks after completion of the last cycle of neoadjuvant treatment. All surgical procedures, including the extent of gastric resection and D2 lymph node dissection, are performed according to the guidelines of the Japanese Research Society for the Study of Gastric Cancer.

Adjuvant treatment

Adjuvant treatment started 3 to 8 weeks after gastrectomy. Patients will receive five 3-week cycles of adjuvant treatment with CA-SAP or SAP according to the preoperative regimen.

Statistical Consideration

Analysis sets

Modified intention-to-treat (mITT) set included all patients who are randomly assigned and received at least one dose of allocated treatment. This population will be used for the efficacy analyses.

Per-protocol set included patients in the mITT population who did not present major deviations from the protocol. We pre-specified that participants who received surgery after administration of neoadjuvant CA-SAP or SAP are included in this set. This population will be also used for the efficacy analyses.

Safety analysis set included patients who received at least one dose of allocated treatment. This population will be also used for the safety analyses.

Sample size determination

Based on the assumption of MPR rates of 15% in the SAP group and 35% in the CA-SAP group, a sample size of 53 patients per group

	was required to detect improvement with 80% power and a one-sided alpha level of 0.1 (Fisher's exact test), including a 5% dropout rate.
**Version**	1.3

Study Plan

Items	Screening Period		Randomization	Neoadjuvant Treatment Period					Surgery		Adjuvant Treatment Period		Follow-up[15]
	Before 2 weeks of enrollment	Before 1 week of enrollment		Cycle 1	Cycle 2		Cycle 3		Before 1 week of surgery	After 1 week of surgery	Cycle 1-5		
				Day 8 (±3)	Day 1 (±3)	Day 8 (±3)	Day 1 (±3)	Day 8 (±3)			Day 1 (±3)	Day 8 (±3)	
**Baseline characters**													
Informed consent	Prior to randomization												
Demographics data	×												
Medical history	×												
Vital signs and physical		×		×	×	×	×	×	×	×	×	×	×
**Assessments**													
Blood routine[2]		×		×	×	×	×	×	×	×	×	×	×
Urinary routine[3]		×			×		×		×		×		
Fecal routine[4]		×			×		×		×		×		
Biochemistry[5]		×		×	×	×	×	×	×	×	×	×	×
Coagulation test[6]		×			×		×		×		×		
Thyroid function test[7]		×			×		×		×		×		
Tumor markers[8]		×			×		×		×		×		×
HBV/HCV/HIV[9]		×							×				
Tumor sample[10]		×								×			
Gastroduodenoscopy		×											
12-lead ECG		×			×		×		×		×		
Pregnancy test[11]		×											

**Imaging examination[12]**	×						×		×				×
**ECOG status**		×			×		×		×	×	×		
**Blood pressure monitoring[13]**		×		×	×	×	×	×	×	×	×	×	
**Adverse events[14]**				×	×	×	×	×	×	×	×	×	
**Others**													
**Concomitant treatments**	×			×	×	×	×	×	×	×	×	×	
**Drug compliance**					×		×		×		×		

[1] Including vital signs (blood pressure, pulse, and temperature), height (at baseline only), weight, and physical examination (especially abdominal examination).

[2] Including hemoglobin, hematocrit, RBC, WBC, ANC, and platelet count.

[3] Including urinary protein and urine occult blood.

[4] Including fecal occult blood. Gastroduodenoscopy should be performed if the patient has persistent OB (+) before resection of primary tumor.

[5] Including bilirubin, aspartate aminotransferase (AST), alanine aminotransferase (ALT), alkaline phosphatase (ALP), creatinine, blood urea nitrogen (BUN), glucose, calcemia, sodium, and potassium.

[6] Including prothrombin time (PT), activated partial thromboplastin time (APTT), thrombin time (TT), and fibrinogen.

[7] Including thyroid-stimulating hormone (TSH), free T3, and free T4.

[8] Including carcinoembryonic antigen (CEA) and carbohydrate antigen (CA)19-9.

[9] Including hepatitis B virus (HBV), hepatitis C virus (HCV), and human immunodeficiency virus (HIV).

[10] Central evaluation of programmed death-ligand 1 (PD-L1) expression and microsatellite instability (MSI) in pretreatment specimens, and tumor regression grade in surgical specimens.

[11] For women of child-bearing age.

[12] Including chest, abdomen and pelvis CT or MRI.

[13] Blood pressure monitoring is completed by the patient and recorded in a diary card. Blood pressure should be measured at least 3 times a week for the first 2 cycles. For patients with abnormal blood pressure, blood pressure should be measured every day. At each visit, blood pressure should be measured by the investigator. Each blood pressure measurement should be taken ipsilateral.

[14] Adverse events are recorded from randomization until 30 days after the last dose of the investigational product. During follow-up, all adverse events that occur previously and are ongoing, and newly occurring adverse events related with the investigational product or determined by the investigator as having a reasonable possibility of being caused by the investigational product are recorded, and of these, serious adverse events are all reported. Serious adverse events ongoing at the time of completion of the investigational product administration will be followed during the follow-up period until resolved or stabilized irrespective of the relationship with the investigational product.

[15] Patients are followed up postoperatively by physical examination, laboratory tests, and imaging examination every 3 months for 2 years, every 6 months during years 3-5, and annually thereafter. Endoscopy is recommended annually.

1. Background

There were over 1,000,000 new cases and an estimated 783,000 deaths caused by gastric cancer (GC) in 2018, making it the fifth most frequently diagnosed cancer and the third leading cause of cancer death [1]. It is a serious threat to human health and brings a heavy economic burden to families and society. As the National Central Cancer Registry reported, there were approximately 410,000 new cases of GC and 290,000 deaths every year in China [2]. With the improvement of diagnosis and treatment methods and deepening understanding of the molecular mechanism, the incidence and mortality of GC have shown a downward trend. However, it still faces great challenges.

Endoscopic or surgical resection is curative in most early gastric cancers, with a 5-year overall survival (OS) rate greater than 90% [2]. However, there are no specific signs with early GC, and most patients are diagnosed at advanced stages at first admission [3]. Compared with early GC, advanced GC often causes invasion and adhesion to surrounding tissues and sheds tumor cells into the blood, leading to a low radical resection rate [4]. Disease recurrence occurs in over 50% of patients even after the complete dissection of the primary tumors and regional lymph nodes due to micrometastases [3]. To improve the prognosis of patients with advanced GC, the treatment mode has changed from “surgery alone” to “neoadjuvant chemotherapy + surgery + adjuvant chemotherapy” [4].

Neoadjuvant chemotherapy was first introduced in the multimodal treatment of GC in 1989 by Wilke et al. [5]. In this study, advanced GC patients were treated with EAP regimen. Among them, 33 patients underwent R0/R1 surgical resection and postoperative chemotherapy, with a response (CR+PR) rate of 70%. A meta-analysis by Paoletti et al. showed that the 5-year survival rate was improved by 5.7% in patients undergoing neoadjuvant chemotherapy plus surgery than in those undergoing surgery alone [6]. The MAGIC phase 3 trial published in 2006 demonstrated that perioperative

chemotherapy with epirubicin, cisplatin, and fluorouracil (ECF) could increase the 5-year OS rate from 23% to 36% compared with surgery alone [7]. The FNCLCC and FFCD trial also demonstrated that perioperative chemotherapy using fluorouracil plus cisplatin significantly increased the R0 resection rate, disease-free survival (DFS), and OS [8]. In the FLOT4 trial published in 2019, OS and DFS were significantly improved in the FLOT group (fluorouracil, leucovorin, oxaliplatin, and docetaxel) compared with the ECF/ECX group (epirubicin, cisplatin, and fluorouracil) [9]. Based on these results, the FLOT has become a new standard perioperative regimen in Europe. In China, neoadjuvant chemotherapy using platinum and fluorouracil with or without paclitaxel has been recommended as the first-line treatment for LAGC [10].

Although neoadjuvant chemotherapy has been widely accepted in both Eastern and Western countries, there is still no consensus on the optimal chemotherapy regimen. In recent years, paclitaxel-based chemotherapy has proven efficacy in GC [9,11-12]. Nanoparticle albumin bound (nab)-paclitaxel is a 130 nm particle formulation consisting of paclitaxel and albumin nanoparticles linked by a non-covalent bond, which minimizes the risk of hypersensitivity reactions without premedication. Additionally, nab-paclitaxel exhibits distinct biodistribution properties and increased antitumor efficacy compared with solvent-based paclitaxel [13-14]. In vitro, nab-paclitaxel showed stronger antitumor activity on human gastric cancer cell lines than oxaliplatin and epirubicin [15].

In the past decade, several clinical trials have been performed to investigate molecular targeted therapy for GC, but few molecular agents have shown promising activity [16-18]. The ToGA trial demonstrated that trastuzumab with cisplatin and capecitabine or 5-FU (XP/FP) was associated with improved OS for HER2-positive advanced GC [19]. Unfortunately, only a small percentage of patients (approximately 15%) are ideal candidates for HER-2 targeted therapy. Another well-established target is vascular endothelial growth factor (VEGF). VEGF is one of the most potent angiogenic factors and is a signaling molecule secreted by many solid tumors [20]. Since high VEGF expression is one of the characteristic features of gastric carcinomas, targeting VEGF is therefore considered a promising therapeutic strategy. Apatinib, a

novel receptor tyrosine kinase inhibitor selectively targeting VEGF receptor 2 (VEGFR-2), strongly inhibited VEGF-mediated endothelial cell migration, proliferation, and tumor microvascular density [21]. A phase III study showed that apatinib improved OS and PFS in patients with chemotherapy-refractory advanced or metastatic GC when compared with placebo [22]. In our earlier study, apatinib combined with chemotherapy was effective as neoadjuvant treatment for LAGC, with a MPR rate of 25.0%, and had an acceptable safety profile [23].

Host immunity is fundamental to the suppression of human cancer, and conversely host immune evasion by tumor cells is an essential feature in the development and progression of human cancer. Programmed cell death protein 1 (PD-1) is a co-inhibitory receptor expressed on the surface of activated and exhausted T-cells, B-cells, and certain myeloid cells [24]. PD-L1, one of two ligands for PD-1, is highly expressed in certain human tumors and expression has been associated with a poor prognosis [25-26]. PD-1 inhibitor, which suppresses the interaction between the PD-1 and its ligands, has demonstrated encouraging antitumor activity in advanced GC, particularly in combination with chemotherapy. In the KEYNOTE-061 study, pembrolizumab did not significantly improve OS or PDS compared with paclitaxel [27]. In contrast, pembrolizumab exhibited a higher ORR (60.0% vs. 25.8%) in combination with cisplatin and fluorouracil compared with its monotherapy in the KEYNOTE-059 study [28].

Multiple preclinical models have supported the synergistic effects between angiogenic inhibitors and PD-1 inhibitors [29-30]. In the IMmotion151 trial, atezolizumab (anti－PD-L1) + bevacizumab (anti-VEGF) showed longer PFS compared with sunitinib in patients with metastatic renal cell carcinoma [31]. These findings suggest a strong rationale for combining PD-1 inhibitors and angiogenic inhibitors. Camrelizumab, a high-affinity humanized IgG4 monoclonal antibody targeting PD-1, has shown clinically significant efficacy in advanced GC [32]. Thirty patients with chemotherapy-refractory recurrent or metastatic GC were enrolled. Of these, 7 patients (23.3%) demonstrated objective responses, including 1 CR. The ORRs for patients with PD-L1–positive and PD-L1–negative tumors were 23.1% (3 of 13) and

26.7% (4 of 15), respectively (P = 1.000). Two treatment-related grade 3 or higher adverse events were reported: one was grade 3 pruritus, and the other (3.3%) was grade 5 interstitial lung disease. We hypothesized that camrelizumab and apatinib combined with chemotherapy might be beneficial in patients with LAGC. Thus, we conducted a phase 2 trial (Arise-FJ-G005) to investigate the efficacy and safety of camrelizumab and apatinib combined with nab-paclitaxel plus S-1 versus nab-paclitaxel plus S-1 alone as neoadjuvant treatment for LAGC.

2. Objectives

2.1 Primary Objective

To assess the efficacy of camrelizumab and apatinib combined with nab-paclitaxel plus S-1 versus nab-paclitaxel plus S-1 alone as neoadjuvant treatment for LAGC, as measured by MPR according to the Becker regression criteria.

2.2 Secondary Objectives

To assess and compare the followings between the two groups:

- Radiologic response according to the RECIST, version 1.1
- R0 resection rate
- Safety of study drugs according to the CTCAE, version 5.0
- Surgical safety: including morbidity, mortality, hospital stay, etc.
- OS and PFS

2.3 Exploratory Objective

To assess the associations between the primary endpoint and several tumor biomarkers (including but not limited to PD-L1 expression and MSI status)

3. Study Design and Sample Size

3.1 Study Design

This is a multicenter, randomized, open-label, phase 2 trial to investigate the efficacy and safety of camrelizumab and apatinib combined with nab-paclitaxel plus S-1 versus nab-paclitaxel plus S-1 alone as neoadjuvant treatment for LAGC. It will be conducted in 5 medical centers in China. Eligible patients were randomly assigned to receive camrelizumab (200 mg intravenously on day 1) and apatinib (250 mg orally once daily on days 1 to 21) combined with chemotherapy (nap-paclitaxel 125 mg/m^2 intravenously on days 2 and 9, S-1 40 to 60 mg orally twice daily depending on BSA on days 1 to 14) or chemotherapy alone every 3 weeks for 3 preoperative cycles followed by 5 postoperative cycles. All patients will be followed for survival.

3.2 Study Schema

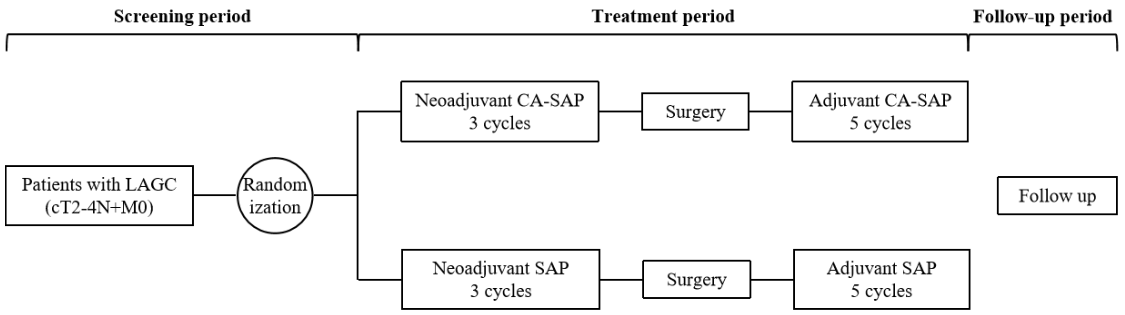

3.3 Study Duration

The total duration for enrollment is expected to be 24 months, beginning with the first patient in June, 2019, and ending with the last patient in June, 2022. The initial analyses for the primary endpoint are planned in the third quarter of 2022. The total duration of the study is expected to be 5 years, including a follow-up period of 3 years. The final analyses for OS and PFS are planned in the third quarter of 2025.

3.4 Sample Size

Based on the assumption of MPR rates of 15% in the SAP group and 35% in the CA-SAP group, a sample size of 53 patients per group is required to detect improvement with 80% power and a one-sided alpha level of 0.1 (Fisher's exact test), including a 5% dropout rate.

4. Study Population

4.1 Inclusion Criteria

- Age older than 18 and younger than 75 years
- Primary gastric adenocarcinoma confirmed pathologically by endoscopic biopsy
- Clinical stage T2-4aN+M0 disease as assessed by CT/MRI, PET-CT, and laparoscopy, if feasible
- At least one measurable lesion according to the RECIST, version 1.1
- ECOG performance status of 0 or 1
- Life expectancy of at least 12 weeks
- Acceptable bone marrow, hepatic, and renal function, including:

blood routine examination (No blood transfusion within 14 days)

White blood cell count >3.0 × 10^9 cells per L and platelet count >100 × 10^9 cells per L

Total bilirubin <1.5 × ULN, alanine aminotransferase or aspartate amino transferase <2.5 × ULN

Serum creatinine ≤1 × ULN, or calculated glomerular filtration rate >60 mL/min

- Written informed consent

4.2 Exclusion Criteria

- Prior chemotherapy, radiotherapy, targeted therapy, or immunotherapy
- Contraindications for surgical treatment or chemotherapy
- Presence of distant metastasis
- History of other malignant disease within the past 5 years, except: basal cell carcinoma of the skin, squamous cell carcinoma of the skin, or in situ cervical cancer
- Any active or history of autoimmune disease, or history of syndrome that required

systemic steroids or immunosuppressive medications, except for subjects with vitiligo or resolved childhood asthma/atopy

- History of immunodeficiency diseases, including human immunodeficiency virus (HIV), or other acquired or congenital immune-deficient disease, or transplantation
- Severe heart, lung, liver, or renal dysfunction
- Severe mental disorder
- Presence of digestive tract obstruction, jaundice, or acute infectious diseases
- Pregnant or breast-feeding women
- Uncontrolled blood pressure on medication (>140/90 mmHg)
- Evidence of bleeding tendency or receiving thrombolytics or anticoagulants
- History of interstitial lung disease or non-infectious pneumonia
- Positive urinary protein (urinary protein ≥2+ or 24h urine protein >1 g)
- Sensitivity to study drugs

4.3 Drop Out/Removal Criteria

- Use of NMPA-approved modern traditional Chinese medicine agents for the treatment of gastric cancer
- Concurrent radiotherapy or other local treatments during the study periods
- Medication, dosing, and treatment methods do not follow the study protocol

4.4 Discontinue Criteria

- Withdrawn of the informed consent
- PD
- Unbearable toxicity after dose reductions
- Pregnancy
- Loss to follow up
- Investigator’s decision that stopping treatment is in the best interest of the patient
- Death

5. Study Treatments

5.1 Handling and Storage

Study drugs must be stored in a secure area under the appropriate physical conditions. Access to and administration of the study drugs will be limited to the investigators and authorized site staff. The investigators must complete storage, handling, dispensing, and infusion information for the study drugs in time.

5.2 Treatment

The study drugs that are used in this trial are outlined below in **Table 1**.

Table 1. Study drugs.

**Product name**	Camrelizumab	Apatinib	Nap-paclitaxel	S-1
**Dosage form**	Lyophilized powder	Tablet	Lyophilized powder	Capsule
**Unit dose strength**	200 mg	250 mg	100 mg	20 mg
**Route of administration**	IV infusion	Oral	IV infusion	Oral
**Storage requirements**	2-8°C. Protect from light and freezing.	<25°C. Protect from light.	20-30°C. Protect from light.	<25°C. Protect from light.

Treatment will be assigned to eligible patients after signed consent forms:

- Camrelizumab is to be administered initially at 200 mg as a 30-minute IV infusion every 3 weeks for 3 preoperative cycles followed by 5 postoperative cycles.
- Apatinib is to be administered initially at 250 mg orally once daily on days 1 to 21 every 3 weeks for 3 preoperative cycles followed by 5 postoperative cycles.
- Nap-paclitaxel is to be administered initially at 125 mg/m^2 as a 30-minute IV infusion every 3 weeks for 3 preoperative cycles followed by 5 postoperative cycles.
- S-1 is to be administered initially at 40-60 mg orally twice daily on days 1 to 14 every 3 weeks for 3 preoperative cycles followed by 5 postoperative cycles.

Table 2. Dose of S-1 depending on BSA.

**BSA**	**Dose**
< 1.25m^2	40mg× 2/day

1.25m^2 - 1.5m^2	50mg× 2/day
> 1.5m^2	60mg× 2/day

5.3 Dose Modification

Dose modification will be performed based on the severity of toxicities according to the CTCAE, version 5.0. Reasons for dose interruption, delay, or reduction, the supportive measures, and the outcomes will be documented. Dose interruptions are permitted for reasons apart from toxicities, such as medical/surgical events or logistical reasons (e.g., unrelated medical events or elective surgery).

For participants treated with CA-SAP, if a toxicity is considered to be due solely to one investigational agent (i.e., camrelizumab, apatinib, or chemotherapeutic agents), the dose of that agent should be interrupted or delayed in accordance with the guidelines below and the treatment with other agents can continue as scheduled at the discretion of the investigator. When the attribution of toxicity is uncertain, interruption of apatinib and chemotherapeutic agents is done first. If one agent is discontinued due to unacceptable toxicity, patients are able to continue the study with the other agent.

5.3.1 Dose Modification for Camrelizumab

Toxicities associated or possibly associated with camrelizumab may represent an immunologic etiology. For suspected immune-related AEs (irAEs), ensure adequate evaluation to confirm etiology or exclude other causes.

In general, administration of camrelizumab may be continued in the presence of most grade 1 irAEs. For grade 2-4 irAEs, camrelizumab is usually discontinued and can be resumed once irAEs resolve to ≤grade 1. Camrelizumab should be permanently discontinued if the irAE cannot resolve within 12 weeks of the last dose or corticosteroids cannot be reduced to ≤10 mg/day prednisone or equivalent within 12 weeks. Permanent discontinuation of camrelizumab should be considered for any severe or life-threatening events.

The use of corticosteroids is the mainstay of management of irAEs. For severe and

life-threatening irAEs, corticosteroids should be initiated intravenously first followed by oral administration. With improvement to ≤grade 1, initiate corticosteroid taper and continue to taper over at least 1 month. Other immunosuppressive treatment should be initiated if irAEs cannot be controlled by corticosteroids within 3-5 days.

Dose reduction is not permitted for camrelizumab.

- **Immune-mediated Pneumonitis**

Camrelizumab can cause immune-mediated pneumonitis, defined as requiring use of corticosteroids and no clear alternate etiology.

Patients should be monitored for signs with radiographic imaging and for symptoms of pneumonitis. Administer corticosteroids at a dose of 1 mg/kg/day prednisone equivalents for grade 2 pneumonitis. Administer corticosteroids at a dose of 2-4 mg/kg/day prednisone equivalents followed by corticosteroid taper for grade 3-4 pneumonitis. Withhold camrelizumab for grade 2 pneumonitis until resolution for ≤grade 1 pneumonitis. Permanently discontinue camrelizumab for grade 3-4 pneumonitis.

- **Immune-mediated Hepatitis**

Camrelizumab can cause immune-mediated hepatitis, defined as requiring use of corticosteroids and no clear alternate etiology.

Patients should be monitored for abnormal liver tests prior to and periodically during treatment. Administer corticosteroids at a dose of 0.5-1 mg/kg/day prednisone equivalents for grade 2 transaminase elevations. Administer corticosteroids at a dose of 1-2 mg/kg/day prednisone equivalents followed by corticosteroid taper for grade 3-4 transaminase elevations, with or without concomitant elevation in total bilirubin. Withhold camrelizumab for grade 2-3 pneumonitis until resolution for ≤grade 1 pneumonitis. Permanently discontinue camrelizumab for grade 4 or for recurrent transaminase elevations upon re-initiation of camrelizumab.

- **Immune-mediated Colitis**

Camrelizumab can cause immune-mediated colitis, defined as requiring use of corticosteroids with no clear alternate etiology.

Patients should be monitored for signs and symptoms of colitis. Administer corticosteroids at a dose of 0.5-1 mg/kg/day prednisone equivalents followed by corticosteroid taper for grade 2 colitis of more than 5 days duration; if worsening or no improvement occurs despite initiation of corticosteroids, increase dose to 1-2 mg/kg/day prednisone equivalents. Administer corticosteroids at a dose of 1-2

mg/kg/day prednisone equivalents followed by corticosteroid taper for grade 3-4 colitis. In cases of corticosteroid-refractory colitis, consider repeating infectious workup to exclude alternative etiologies. Addition of an alternative immunosuppressive agent to the corticosteroid therapy, or replacement of the corticosteroid therapy should be considered in corticosteroid-refractory immune-mediated colitis if other causes are excluded. Withhold camrelizumab for grade 2-3 colitis until resolution for ≤grade 1 colitis. Permanently discontinue camrelizumab for grade 4 or for recurrent colitis upon re-initiation of camrelizumab.

- **Immune-mediated Endocrinopathies**

<u>Hypothyroidism and Hyperthyroidism</u>

Camrelizumab can cause autoimmune thyroid disorders.

Monitor thyroid function prior to and periodically during camrelizumab treatment. Administer hormone-replacement therapy for hypothyroidism. Initiate medical management for control of hyperthyroidism. Withhold camrelizumab for grade 3 hypothyroidism until initiation of hormone-replacement therapy. Withhold camrelizumab for grade 3 hyperthyroidism until resolution for ≤grade 2 hyperthyroidism. Permanently discontinue camrelizumab for grade 4 hypothyroidism or hyperthyroidism.

<u>Others</u>

Other endocrinopathies include hypophysitis, adrenal insufficiency, type 1 diabetes mellitus, etc. Monitor patients for signs and symptoms. Withhold camrelizumab for grade 2-3. Permanently discontinue camrelizumab for grade 4 endocrinopathies.

- **Immune-Mediated Nephritis and Renal Dysfunction**

Camrelizumab can cause immune-mediated nephritis, defined as renal dysfunction or ≥ grade 2 increased creatinine, requirement for corticosteroids, and no clear alternate etiology.

Monitor patients for elevated serum creatinine prior to and periodically during treatment. Administer corticosteroids at a dose of 0.5-1 mg/kg/day prednisone equivalents for grade 2-3 increased serum creatinine, if worsening or no improvement occurs, increase dose of corticosteroids to 1-2 mg/kg/day prednisone equivalents. Administer corticosteroids at a dose of 1-2 mg/kg/day prednisone equivalents followed by corticosteroid taper for grade 4 increased serum creatinine. Withhold camrelizumab for moderate grade 2 increased serum creatinine until resolution for ≤grade 1 increased serum creatinine. Permanently discontinue camrelizumab for grade 3-4 increased serum

creatinine.

- **Immune-mediated Skin Adverse Reactions**

Camrelizumab can cause immune-mediated rash, including reactive cutaneous capillary endothelial proliferation (RCCEP).

Patients should be monitored for signs and symptoms. Administer corticosteroids at a dose of 1 to 2 mg/kg/day prednisone equivalents followed by a corticosteroid taper for grade 3-4 rash. Withhold camrelizumab for grade 3 rash until resolution for ≤grade 2 rash and permanently discontinue camrelizumab for grade 4 rash.

- **Other Immune-mediated Adverse Reactions**

Camrelizumab can cause other clinically significant and potentially fatal immune-mediated adverse reactions. Immune-mediated adverse reactions may occur after discontinuation of camrelizumab therapy. For any suspected immune-mediated adverse reactions, exclude other causes. Based on the severity of the adverse reaction, permanently discontinue or withhold camrelizumab, administer high-dose corticosteroids, and if appropriate, initiate hormone replacement therapy. Upon improvement to ≤grade 1, initiate corticosteroid taper and continue to taper over at least 1 month. Consider restarting nivolumab after completion of corticosteroid taper based on the severity of the event. Permanently discontinue camrelizumab for severe or life-threatening adverse reactions.

- **Infusion Reactions**

Camrelizumab can cause severe infusion reactions. Discontinue camrelizumab in patients with grade 3-4 infusion reactions. Interrupt or slow the rate of infusion in patients with grade 1-2 infusion reactions.

5.3.2 Dose Modification for Apatinib

Management of treatment-related AEs may require interruption and/or dose reduction of apatinib. The dose reduction levels for apatinib in presence of AEs are listed in **Table 3**. Dose modification of apatinib guidelines are provided in **Table 4**. The dose modifications must be performed as clinically indicated at the discretion of the investigator.

For intolerable grade 2 AEs, apatinib should be interrupted until resolution to ≤grade 1. For grade 3-4 AEs, apatinib should be interrupted until resolution to ≤grade 1 or 2, then resume apatinib with one dose or two dose reductions, or permanently

discontinue treatment which is based on investigator judgement. A maximum of two dose reductions are permitted for apatinib. Permanently discontinue apatinib in the presence of grade 3 or 4 AEs after two dose reductions. However, exception will be permitted in the best interest of the patients. Dose escalation is not permitted.

Table 3. Dose-adjustment criteria for apatinib.

**Level**	**Dose of Apatinib**
Starting dose	250 mg once daily
Decrease 1 dose level	250 mg once daily with two days on and one day off
Decrease 2 dose level	250 mg once daily every other day

Table 4. Dose modification guidelines for apatinib.

**Toxicity**	**Grade**	**Timing for Resumption**	**Dose Modification for Apatinib**
Hematological toxicity	Grade 3	Resolution for ≤grade 2 toxicity	1st occurrence: maintain the same dose.
			2nd occurrence: resume at the next lower dose.
			3rd occurrence: resume at the next lower dose.
	Grade 4	Resolution for ≤grade 2 toxicity	1st occurrence: resume at the next lower dose.
			2nd occurrence: resume at the next lower dose.
Hypertension	Grade 3	Resolution for ≤grade 2 toxicity	1st occurrence: resume at the next lower dose.
			2nd occurrence: resume at the next lower dose.
	Grade 4	Permanently discontinue	Permanently discontinue
Proteinuria	Grade 3	Resolution for ≤grade 2 toxicity	1st occurrence: resume at the next lower dose.
			2nd occurrence: resume at the next lower dose.
Palmar-plantar erythrodysesthesia syndrome	Grade 2 (cannot resolve to ≤grade 1 despite supportive care measures)	Resolution for ≤grade 1 toxicity	1st occurrence: maintain the same dose.
			2nd occurrence: resume at the next lower dose.
			3rd occurrence: resume at the next lower dose.
	Grade 3	Resolution for ≤grade 1	1st occurrence: resume at the next lower dose.

		toxicity	2nd occurrence: resume at the next lower dose.
Fatigue	Grade 2 (cannot resolve to ≤grade 1 despite supportive care measures)	Resolution for ≤grade 1 toxicity	1st occurrence: maintain the same dose.
			2nd occurrence: resume at the next lower dose.
			3rd occurrence: resume at the next lower dose.
	Grade 3	Resolution for ≤grade 1 toxicity	1st occurrence: resume at the next lower dose.
			2nd occurrence: resume at the next lower dose.
Abdominal pain	Grade 2 (cannot resolve to ≤grade 1 despite supportive care measures)	Resolution for ≤grade 1 toxicity	1st occurrence: resume at the next lower dose. 2rd occurrence: resume at the next lower dose.
	Grade 3	Resolution for ≤grade 1 toxicity	
Hemorrhage	Any grade cerebral hemorrhage, grade 2 pulmonary hemorrhage, or grade 3 hemorrhage at any site	Permanently discontinue	Permanently discontinue
Arterial thromboembolism	Any grade	Permanently discontinue	Permanently discontinue
Pulmonary fibrosis	Any grade	Permanently discontinue	Permanently discontinue
Other non-hematological toxicities	Grade 3 or 4	Resolution for ≤grade 1 toxicity	1st occurrence: resume at the next lower dose.
			2nd occurrence: resume at the next lower dose.

5.3.3 Dose Modification for Chemotherapeutic Agents

For intolerable grade 2 AEs, chemotherapeutic agents (nap-paclitaxel and S-1) should be interrupted until resolution to ≤grade 1. For grade 3-4 AEs, chemotherapeutic agents

should be interrupted until resolution to ≤grade 1 or 2, then resume treatment with one dose or two dose reductions, or permanently discontinue treatment which is based on investigator judgement. A maximum of two dose reductions are permitted for chemotherapeutic agents. Permanently discontinue chemotherapeutic agents in the presence of grade 3 or 4 AEs after two dose reductions. However, exception will be permitted in the best interest of the patients. Dose escalation is not permitted. Dose modification of chemotherapeutic agents guidelines are provided in **Table 5**. The dose modifications must be performed as clinically indicated at the discretion of the investigator.

Table 5. Dose modification guidelines for chemotherapeutic agents.

**Toxicity**	**Grade**	**Timing for Resumption**	**Dose Modification for Chemotherapeutic Agents**
Hematological toxicity	Grade 3-4	Resolution for ≤grade 1 toxicity	1st occurrence:<br>75% of Nap-paclitaxel starting dose<br>75% of S-1 starting dose
			2nd occurrence:<br>50% of Nap-paclitaxel starting dose<br>50% of S-1 starting dose
Mucositis/stomatitis, diarrhea, and anorexia	Grade 2	Resolution for ≤grade 1 toxicity	1st occurrence:<br>maintain the same dose
			2nd occurrence:<br>75% of S-1 starting dose
			3rd occurrence:<br>50% of S-1 starting dose
	Grade 3	Resolution for ≤grade 1 toxicity	1st occurrence:<br>75% of S-1 starting dose
			2nd occurrence:<br>50% of S-1 starting dose
	Grade 4	Resolution for ≤grade 1 toxicity	1st occurrence:<br>50% of S-1 starting dose or permanently discontinue
Peripheral neuropathy	Grade 2 (cannot resolve to ≤grade 1 despite supportive care measures)	Resolution for ≤grade 1 toxicity	1st occurrence:<br>75% of Nap-paclitaxel starting dose<br>2nd occurrence:<br>50% of Nap-paclitaxel starting dose
	Grade 3-4	Resolution for ≤grade 1 toxicity	
Other non-hematological toxicity	Grade 2	Resolution for ≤grade 1	1st occurrence:<br>maintain the same dose

		toxicity	2nd occurrence:<br>75% of Nap-paclitaxel starting dose<br>75% of S-1 starting dose
			3rd occurrence:<br>50% of Nap-paclitaxel starting dose<br>50% of S-1 starting dose
	Grade 3	Resolution for ≤grade 1 toxicity	1st occurrence:<br>75% of Nap-paclitaxel starting dose<br>75% of S-1 starting dose
			2nd occurrence:<br>50% of Nap-paclitaxel starting dose<br>50% of S-1 starting dose
	Grade 4	Resolution for ≤grade 1 toxicity	1st occurrence:<br>50% of Nap-paclitaxel and S-1 starting dose or permanently discontinue

5.4 Concomitant Therapy

Concomitant therapy refers to any type of treatment continued from 14 days before randomization, during the study administration period, and until 30 days after the last dose, or all treatment initiated since the first dose of study drugs. Such treatment should be carefully and accurately documented in the Case Report Forms (CRFs).

5.5 Prudently or Forbidden Used Drug

Camrelizumab is a human monoclonal antibody that is not metabolized by cytochrome P450 enzymes or other drug metabolizing enzymes. These are not expected to have pharmacokinetic-based interactions.

Apatinib has a strong inhibition activity on CYP3A4, YP2C9 and CYP2C19 (IC50 <0.5 μM). The CYP3A4 inductor (Dexamethasone, catarrh imipramine, Rifampin, and Phenobarbital), inhibitors (Ketoconazole, Itraconazole, Erythromycin, and Klaricid) and substrates (Simvastatin, Cyclosporine and Pimozide) should be used with caution. Other drugs metabolized by CYP3A4 (such as Benzodiazepines, Dihydropyridine, calcium channel blockers, and HMG-COA reductase inhibitors) and CYP2C9, CYP2C19 substrates should also be used with caution.

6. Study Procedure

6.1 Screening Period

The following items are completed within 2 weeks before randomization:

- Demographics data: age, gender, date of birth, height, weight, etc.
- Tumor data: date of diagnosis, histological classification, clinical stage, etc.
- Medical history: comorbidities, previous medication/surgery, etc.
- Imaging examination: chest, abdomen and pelvis CT or MRI.

After admission, patients who meet the eligibility criteria are screened out. For these patients, the investigator introduces this trial in detail, and the patients sign informed consent if they agree to participate.

The following items are completed within 1 week before randomization:

- ECOG performance status.
- Vital signs: heart rate, breathing rate, temperature, and blood pressure.
- Physical examination: head, skin, lymph nodes, eyes, ears, nose, throat, mouth, respiratory system, cardiovascular system, abdomen, genitourinary system, musculoskeletal system, and nervous system.
- Blood routine: hemoglobin, hematocrit, RBC, WBC, ANC, and platelet count.
- Urinary routine: a 24-hour urinary protein quantification test should be performed in the presence of urine protein ≥2+.
- Stool routine: fecal occult blood.
- Biochemistry: bilirubin, ALT, AST, ALP, creatinine, glucose, calcemia, sodium, and potassium.
- Coagulation test: PT, APTT, TT, and fibrinogen.
- Thyroid function test: TSH, fT3, and fT4.
- Tumor markers: CEA and CA 19-9.
- HBV, HCV, and HIV tests.
- Pretreatment tumor samples.
- 12-lead ECG.
- Pregnancy tests (for women at childbearing age).

6.2 Treatment Period

6.2.1 Neoadjuvant Treatment Period

Administration of the neoadjuvant treatment is initiated within 7 days after randomization. The following items are completed during the neoadjuvant treatment:

- Physical examination, vital signs, ECOG performance status, tumor markers, and ECG are performed on day 1 of cycles 2-3. If cardiac pains or palpitations occur, myocardial enzyme spectrum (creatine kinase, lactate dehydrogenase), ECG, and cardiac ultrasound should be immediately performed.
- Blood routine and biochemistry are performed on day 8 of cycle 1 and days 1, 8 of cycles 2-3.
- Urinary routine, stool routine, coagulation test, thyroid function test, and tumor markers are performed on day 1 of cycles 2-3.
- Imaging examination is performed after completion of cycle 2.
- AEs during the treatment period should be recorded and graded according to the CTCAE, version 5.0.
- Blood pressure monitoring is performed by the patient and recorded in a diary card.

6.2.2 Preoperative Assessments and Surgery

After completion of the last cycle of neoadjuvant treatment, blood routine, biochemistry, stool routine, urine routine, 12-lead ECG, coagulation test, thyroid function test, and tumor markers are performed. Without clear surgical contraindications and with the patients' consent, surgery will be performed 2-4 weeks later. If the disease progresses, laparoscopic exploration is recommended. The following items are completed after surgery:

- Surgical outcomes: operation time, blood loss, intraoperative complications (e.g. major bleeding), postoperative complications and management, postoperative hospital stay, etc.
- Tumor data: tumor regression grade, residual tumor (R) classification, etc.
- Surgical specimens.

6.2.3 Adjuvant Treatment Period

Adjuvant treatment started 3-8 weeks after gastrectomy. The following items are completed during the adjuvant treatment:

- Physical examination, vital signs, ECOG performance status, tumor markers, and

ECG are performed on day 1 of cycles 2-5. If cardiac pains or palpitations occur, myocardial enzyme spectrum (creatine kinase, lactate dehydrogenase), ECG, and cardiac ultrasound should be immediately performed.

- Blood routine and biochemistry are performed on day 8 of cycle 1 and days 1, 8 of cycles 2-5.
- Urinary routine, stool routine, coagulation test, thyroid function test, and tumor markers are performed on day 1 of cycles 2-5.
- Imaging examination is performed every 3 months.
- AEs during the treatment period should be recorded and graded according to the CTCAE, version 5.0.
- Blood pressure monitoring is performed by the patient and recorded in a diary card.

6.3 Follow-up Period

After completion of the last administration of study drugs, patients enter follow-up period. All patients are followed up with every 3 months (± 2 weeks) during the first 2 years and then every 6 months (± 2 weeks) beyond the third year. Data on survival status and disease progression are recorded in the follow-up tables.

Assessments during the follow-up period include physical examination, blood routine, biochemistry, tumor markers, abdominal ultrasound, and chest, abdomen and pelvis CT. Gastroscopy is recommended to performed once a year.

6.4 Progression of Disease

If any of the following criteria is met, it is defined as the progressive disease:

- Determination as PD according to the RECIST, version 1.1 during the neoadjuvant treatment
- Reporting of a distant metastasis from pathology
- R1 or R2 resection
- Finding of a recurrence/distant metastasis during follow-up after R0 resection

For a subject determined to have disease progression, administration of the study drugs will be discontinued and other anti-tumor treatment will be administered at the discretion of the investigator.

7. Efficacy Assessments

- **Pathological Response**

Tumor regression grade (TRG) is evaluated centrally using the Becker regression criteria, which are based on the percentage of vital tumor cells in the tumorous area and include the following categories: TRG 1a (no residual tumor cells), TRG 1b (<10% residual tumor cells), TRG 2 (10-50% residual tumor cells) and TRG 3 (>50% residual tumor cells). The MPR rate is defined as the proportion of patients with <10% residual tumor cells in resection specimens (TRG 1a/1b). More details are provided in **Appendix 1**.

- **Radiologic Response**

Radiologic response is evaluated using RECIST (version 1.1), which is based on CT or MRI findings and includes complete response (CR), partial response (PR), stable disease (SD), and progressive disease (PD). The objective response rate (ORR) was defined as the proportion of patients with CR and PR, and the disease control rate (DCR) was defined as the proportion of patients with CR, PR, and SD. More details are provided in **Appendix 4**.

In a comparison between the pretreatment and posttreatment clinical staging, T downstaging, N downstaging are compared between the two groups based on the CT findings.

- **Surgical Margin**

Tumor condition is explained according to the residual tumor (R) classification:

- R0: No residual cancer (negative cross-section)
- R1: Microscopically observed residual cancer (positive cross-section)
- R2: Macroscopically observed residual cancer

R0 resection rate is defined as the proportion of patients who achieve the R0 resection.

- **Survival**

OS is defined as the time between randomization and the death date. Patients alive at last report will be considered censored at the endpoint. Alive patients will be censored at the last date known to be alive, either during study treatment period or during follow-up period.

PFS is defined as the time between disease progression (see **Section 6.4**) or death irrespective of cause and censored at the date of last contact.

8. Safety Assessments

8.1 Safety Endpoints

Data on all AEs, whether volunteered by the subject, discovered by investigator questioning, or detected through physical examination, laboratory test or other means, will be collected, recorded, and followed as appropriate.

The investigator will collect information on the following variables and record in the CRFs:

- **Surgical safety:** operation time, blood loss, intraoperative complications, postoperative morbidity, mortality, postoperative hospital stay, time to ambulation, time to first flatus, and time to first liquid intake.

- Postoperative morbidity is evaluated according to the Clavien-Dindo classification (**Appendix 6**)

- **Study drug safety:** AEs and serious AEs (SAEs)

- All AEs should be recorded by it duration, regulatory seriousness criteria, suspected relationship to the study drug, and actions taken. AEs are evaluated according to the CTCAE, version 5.0.

- **Details on drug modification caused by AEs**

8.2 Definition of Adverse Event and Serious Adverse Event

An AE is defined as any new untoward medical occurrence or worsening of a preexisting medical condition in a clinical investigation participant administered study drug and that does not necessarily have a causal relationship with this treatment. An AE can therefore be any unfavorable and unintended sign (such as an abnormal laboratory finding), symptom, or disease temporally associated with the use of study drugs, whether or not considered related to the study drugs. The causal relationship to study drug is determined by a physician and should be used to assess all AEs.

The **casual relationship** can be one of the following:

- <u>Definitely</u>: An AE which has a timely relationship to the administration of the study drugs, follows a known pattern of response, for which no alternative cause is present.

- Probably: An AE, which has a timely relationship to the administration of the study drugs, follows a known pattern of response, but for which a potential alternative cause may be present.

- Unlikely: An AE which does not have a timely relationship to the administration of the study drugs, follows no known pattern of response, does not reappear or worsen after re-administration of the study drugs (if applicable), and for which there is evidence that it is related to a cause other than the study drugs.

- Unrelated: An AE, for which there is evidence that it is definitely related to a cause other than the study drugs. In general, there is no timely relationship to the administration of the study drugs, or if there is a timely relationship, the event does not follow a known pattern of response, and there is an alternative cause.

A **SAE** is an AE not classified as serious that, at any time, fulfills one or more of the following criteria:

- Results in death
- Is life threatening
- Requires in-patient hospitalization or prolongation of existing hospitalization
- Results in persistent or significant disability/incapacity
- Results in congenital anomaly/birth defect
- Any important medical event that may not be immediately life threatening or result in death or hospitalization but, based upon appropriate medical judgment, may endanger the patient or may require intervention to prevent one of the other outcomes listed in the definition above.
- Suspected transmission of an infectious agent (e.g., pathogenic or nonpathogenic) via the study drug is an SAE.
- Any component of a study endpoint that is considered related to study therapy should be reported as an SAE (e.g. death is an endpoint, if death occurred due to anaphylaxis, anaphylaxis must be reported).

Events initially reported as an AE may also become serious.

8.3 Recording and Follow-up of (Serious) Adverse Events

AEs and SAEs will be recorded from time of signature of informed consent, throughout the treatment period and including 30 days after the last dose of study drugs (SAEs will be collected for 90 days after discontinuation of treatment). During the course of the

study all AEs and SAEs should be followed up for each patient. Every effort should be made to obtain a resolution for all events, even if the events continue after discontinuation or study completion.

The investigator is requested to assess the relationship between the study drugs and the occurrence of each (S)AE. The investigator will use clinical judgment to determine the relationship. Alternatives causes, such as natural history of the underlying diseases, concomitant therapy, other risk factors, and the temporal relationship of the event to the investigational product will be considered and investigated.

All SAEs occurring to any patient on this study, regardless of attribution, should be reported to the Institutional Review Board and regulatory authority within 24 hours of the investigator's knowledge of the events. The report of SAE should be consistent with standard SAE reporting guidelines.

9. Statistical Analysis Plan

9.1 Sample Size Considerations

The primary endpoint of this study is the MPR rate. Based on the assumption of MPR rates of 15% in the SAP group and 35% in the CA-SAP group, a sample size of 53 patients per group is required to detect improvement with 80% power and a one-sided alpha level of 0.1 (Fisher's exact test), including a 5% dropout rate.

9.2 Analysis Sets

- Modified intention-to-treat (mITT) set included all patients who are randomly assigned and received at least one dose of allocated treatment. This population will be used for the efficacy analyses.
- Per-protocol set included patients in the mITT population who did not present major deviations from the protocol. We pre-specified that participants who received surgery after administration of neoadjuvant CA-SAP or SAP are included in this set. This population will be also used for the efficacy analyses.
- Safety analysis set included patients who received at least one dose of allocated treatment. This population will be also used for the safety analyses.

9.3 Statistical Analysis

Baseline characteristics and demographic variables will be summarized in the mITT set. Surgical and pathology results will be summarized in the per-protocol set. Nonsurgical AEs will be summarized in the safety analysis set. Continuous variables are presented as medians and interquartile ranges (IQRs) or means and standard deviations (SDs) and are compared using the Wilcoxon rank sum test or t test. Categorical variables are presented as frequencies and percentages and are compared using the $\chi 2$ test or Fisher's exact test.

The efficacy analysis will be performed using both the mITT and per-protocol sets. The efficacy endpoints, including the MPR rate, R0 resection rate, ORR, DCR, and survival, are compared using the Fisher's exact test. The MPR rate, R0 resection rate, ORR, and DCR will be summarized (i.e., number of patients [%]) and 95% confidence intervals (CIs) for the objective response rate will be provided. Medians and 95% confidence intervals (CIs) for OS and PFS will be calculated. Kaplan-Meier plots of OS and PFS will be presented. Hazard ratio assessment and the corresponding 95% CIs can be presented using the Cox proportional hazard model.

The relationship between biomarkers (including but not limited in PD-L1 expression and MSI status) and the MPR rate will be presented for a subset of patients in the efficacy evaluable population who are available for these biomarkers.

The safety analysis will be done on the safety analysis set. All AEs will be presented as frequencies and percentages in each category. If a patient experiences the same AE multiple times during the treatments, the event will be counted only once and by the greatest severity.

10. Data Management

10.1 CRF Completion Guidelines

For each participant, CRF should be completed by the investigator. The investigator is responsible for ensuring that data recorded in the CRFs are complete, accurate, and legible. The CRF will be served as the original records, and should not be changed. Any correction must not be made on the original records, but an additional narrative can be

used for recording the change, the investigator should sign and date for any change made. All data in the CRFs must come from and be consistent with the source documents, i.e. patient's file or medical records.

The CRFs should be completed before reviewing by the Monitor. For any questionable data in the case report form, the Monitor will issue the Data Request (DRQ) to query the investigator, the investigator should answer it as soon as possible and return.

10.2 Data Lock

After validating the data correction under blinding review, the principle investigator and statistical analysis personnel will lock the data. The locked database is no longer to be changed.

10.3 Quality Control and Quality Assurance

- Investigational personnel must be physicians through training of clinical study and who will work under the guidance of the principal investigator.
- Prior to the study initiation, the pre-study qualification is needed to ensure the facility is equipped with all rescue equipment and meets the standard requirements.
- Recommendations to study nurses who will administer study medications to subjects, have a good understanding of the drug administration, and ensuring the compliance of study subjects.
- Each study center must strictly conduct in accordance with the study protocol, and complete CRFs faithfully.
- The Monitor will monitor the clinical study according to the Good Clinical Practice and the Standard Operating Procedure, ensure the data recording and reporting is correct and intact, all CRFs will be completed correctly and be verified with the original documents, and ensure the study can be conducted according to the study protocol.

11. Ethics and Regulatory Considerations

11.1 Local Regulations / Helsinki Declaration

The study was performed in accordance with the Helsinki Declaration of 1964 and later versions, Guidelines for Good Clinical Practice, and Chinese law.

11.2 Informed Consent

The investigator must explain to subjects that their participation is voluntary and that they may withdraw at any study stage at any time, and will not affect their medical treatment and interest; they also can continue to receive other kind of treatments after withdrawal. Subjects must be informed that their participation and their personal information in the study are confidential. Subjects must be informed of the study nature, the study objectives, anticipated benefits, potential hazards or inconveniences, other available treatments, and subject rights and obligations that meet the Declaration of Helsinki, etc. Subjects should be given sufficient time to consider whether or not they will participate the trial. The signature of the participant is needed on the informed consent form. If new safety information changes the risk/benefit assessments, the informed consent may be modified as necessary. If any modification is made, all subjects (including those who have received treatment) will be notified of the new information and given a revised consent form to continue their participation in the study.

11.3 Independent Ethics Committee/Institutional Review Board

Before the study begins, all participating centers should submit the study protocol and relevant documents (CRFs, consent forms, and other documents that may be required) to the Independent Ethics Committee (IEC)/Institutional Review Board (IRB). The study can only start after obtaining approval from the IEC/IRB. Any amendments to the study protocol must be submitted to the IEC/IRB in accordance with local laws and regulations.

11.4 Confidentiality Agreement

Data obtained by this study is confidential, and disclosure to third parties other than the regulatory authority, the principle investigator, and study personnel is prohibited.

11.5 Record Storage

The investigator should arrange for the storage of the research files until the end of the study. All records and documents pertaining to the conduct of this study and the distribution of the study drugs, including CRFs, consent forms, laboratory test results, and medical inventory records, must be retained by the principal investigator for at least 5 years.

11.6 Study Interruption

The principal investigator can decide to discontinue this study at any time and for any reason; the decision to discontinue the study will be communicated in writing to the participating researchers. Likewise, if other participating researchers decide to withdraw from the study, they must notify the company in writing.

11.7 Protocol Modification

Any modification to the protocol will be recorded in a written revision and signed by the principal investigator. A signed, revised version will be attached to the last version of the protocol.

References

[1] Bray F, Ferlay J, Soerjomataram I, et al. Global cancer statistics 2018: GLOBOCAN estimates of incidence and mortality worldwide for 36 cancers in 185 countries. CA Cancer J Clin 2018; 68: 394-424.

[2] Yang L, Zheng R, Wang N, et al. Incidence and mortality of stomach cancer in China,2014. Chin J Cancer Res 2018; 30: 291-298.

[3] Digklia A, Wagner AD. Advanced gastric cancer: Current treatment landscape and future perspectives. World J Gastroenterol 2016; 22: 2403-2414.

[4] Smyth EC, Nilsson M, Grabsch HI, et al. Gastric cancer. Lancet 2020; 396: 635-648.

[5] Wilke H, Preusser P, Fink U, et al.Preoperative chemotherapy in locally advanced and nonresectable gastric cancer: a phase II study with etoposide, doxorubicin, and cisplatin. J Clin Oncol 1989; 7: 1318-1326.

[6] Paoletti X, Oba K, Burzykowski T, et al.Benefit of adjuvant chemotherapy for resectable gastric cancer: a meta-analysis. JAMA 2010; 303: 1729-1737.

[7] Cunningham D, Allum WH, Stenning SP, et al. Perioperative chemotherapy versus surgery alone for resectable gastroesophageal cancer. N Engl J Med 2006; 355: 11-20.

[8] Ychou M, Boige V, Pignon JR, et al. Perioperative chemotherapy compared with surgery alone for resectable gastroesophageal adenocarcinoma: an FNCLCC and FFCD multicenter phase III trial. J Clin Onco 2011; 29: 1715-1721.

[9] Al-Batran SE, Homann N, Pauligk C, et al. Perioperative chemotherapy with fluorouracil plus leucovorin, oxaliplatin, and docetaxel versus fluorouracil or capecitabine plus cisplatin and epirubicin for locally advanced, resectable gastric or gastro-oesophageal junction adenocarcinoma (FLOT4): a randomised, phase 2/3 trial. Lancet 2019; 393: 1948-1957.

[10] Bu Z, Ji J. Comments on Chinese guidelines for diagnosis and treatment of gastric cancer 2018 (English edition). Chin J Cancer Res 2020; 32: 446-447.

[11] Huang D, Ba Y, Xiong J, et al. A multicentre randomised trial comparing weekly paclitaxel + S-1 with weekly paclitaxel + 5-fluorouracil for patients with advanced gastric cancer. Eur J Cancer 2013; 49: 2995-3002.

[12] Ishigami H, Fujiwara Y, Fukushima R, et al. Phase III Trial Comparing Intraperitoneal and Intravenous Paclitaxel Plus S-1 Versus Cisplatin Plus S-1 in Patients With Gastric Cancer With Peritoneal Metastasis: PHOENIXGC Trial. J Clin Oncol

2018; 36: 1922-1929.

[13] Gradishar WJ, Tjulandin S, Davidson N, et al. Phase III trial of nanoparticle albumin-bound paclitaxel compared with polyethylated castor oil-based paclitaxel in women with breast cancer. J Clin Oncol 2005; 23: 7794-7803.

[14] Guan ZZ, Li QL, Feng FY, et al. Superior efficacy of a Cremophor-free albumin-bound paclitaxel compared with solvent-based paclitaxel in Chinese patients with metastatic breast cancer. Asia Pac J Clin Oncol 2009; 5:165-174.

[15] Zhang C, Awasthi N, Schwarz MA, et al. Superior antitumor activity of nanoparticle albumin-bound paclitaxel in experimental gastric cancer. PLoS One 2013; 8: e58037.

[16] Ryu MH, Yoo C, Kim JG, et al. Multicenter phase II study of trastuzumab in combination with capecitabine and oxaliplatin for advanced gastric cancer. Eur J Cancer 2015; 51: 482-488.

[17] Iqbal S, Goldman B, Fenoglio-Preiser CM, et al. Southwest Oncology Group study S0413: a phase II trial of lapatinib (GW572016) as first-line therapy in patients with advanced, or metastatic gastric cancer. Annals of Oncology 2011; 22: 2610.

[18] Sun W, Powell M, O'Dwyer PJ, et al. Phase II study of sorafenib in combination with docetaxel and cisplatin in the treatment of metastatic or advanced gastric and gastroesophageal junction adenocarcinoma: ECOG5203. J Clin Oncol 2010; 28: 2947-2951.

[19] Ohtsu A, Ajani JA, Bai YX, et al. Everolimus for previously treated advanced gastric cancer: results of the randomized, double-blind, phase III GRANITE-1 study. J Clin Oncol 2013; 31: 3935-3943.

[20] Hicklin DJ, Ellis LM. Role of the vascular endothelial growth factor pathway in tumor growth and angiogenesis. J Clin Oncol 2005; 23: 1011-1027.

[21] Tian S, Quan H, Xie C, et al. YN968D1 is a novel and selective inhibitor of vascular endothelial growth factor receptor-2 tyrosine kinase with potent activity in vitro and in vivo. Cancer Sci 2011; 102: 1374-1380.

[22] Li J, Qin SK, Xu JM, et al. Randomized, Double-Blind, Placebo-Controlled Phase III Trial of Apatinib in Patients With Chemotherapy-Refractory Advanced or Metastatic Adenocarcinoma of the Stomach or Gastroesophageal Junction. J Clin Oncol 2016, 34: 1448-1454.

[23] Lin JX, Xu YC, Lin W, et al. Effectiveness and Safety of Apatinib Plus Chemotherapy as Neoadjuvant Treatment for Locally Advanced Gastric Cancer: A

Nonrandomized Controlled Trial. JAMA Netw Open 2021; 4: e2116240.

[24] Zhang X, Schwartz JC, Guo X, et al. Structural and functional analysis of the costimulatory receptor programmed death-1. Immunity 2004; 20: 337-347.

[25] Dong H, Strome SE, Salomao DR, et al. Tumor-associated B7-H1 promotes T-cell apoptosis: a potential mechanism of immune evasion. Nat Med 2002; 8: 793-800.

[26] Thompson RH, Kuntz SM, Leibovich BC, et al. Tumor B7-H1 is associated with poor prognosis in renal cell carcinoma patients with long-term follow-up. Cancer Res 2006; 66: 3381-3385.

[27] Shitara K, Özgüroğlu M, Bang YJ, et al. Pembrolizumab versus paclitaxel for previously treated, advanced gastric or gastro-oesophageal junction cancer (KEYNOTE-061): a randomised, open-label, controlled, phase 3 trial. Lancet 2018; 392: 123-133.

[28] Bang YJ, Kang YK, Catenacci DV, et al. Pembrolizumab alone or in combination with chemotherapy as first-line therapy for patients with advanced gastric or gastroesophageal junction adenocarcinoma: results from the phase II nonrandomized KEYNOTE-059 study. Gastric Cancer 2019; 22: 828-837.

[29] Huang Y, Yuan J, Righi E, et al. Vascular normalizing doses of antiangiogenic treatment reprogram the immunosuppressive tumor microenvironment and enhance immunotherapy. Proc Natl Acad Sci U S A 2012; 109: 17561-17566.

[30] Schmittnaegel M, Rigamonti N, Kadioglu E, et al. Dual angiopoietin-2 and VEGFA inhibition elicits antitumor immunity that is enhanced by PD-1 checkpoint blockade. Sci Transl Med 2017; 9: eaak9670.

[31] Motzer RJ, Atkins MB, Escudier B, et al. IMmotion151: A Randomized Phase III Study of Atezolizumab Plus Bevacizumab vs Sunitinib in Untreated Metastatic Renal Cell Carcinoma (mRCC). J Clin Oncol 2018; 36:6_suppl, 578-578.

[32] Xu J, Zhang Y, Jia R, et al. Anti-PD-1 Antibody SHR-1210 Combined with Apatinib for Advanced Hepatocellular Carcinoma, Gastric, or Esophagogastric Junction Cancer: An Open-label, Dose Escalation and Expansion Study. Clin Cancer Res 2019; 25: 515-523.

Appendix 1 Becker Regression Criteria

Becker Regression Criteria

Grade	Characteristic
TRG 1a	Complete tumor regression without residual tumor
TRG 1b	<10% residual tumor
TRG 2	10% to 50% residual tumor
TRG 3	50% residual tumor cells

Note: The Becker regression criteria are based on the estimation of the percentage of vital tumor cells in relation to the macroscopically identifiable tumor bed.

Appendix 2 Pathological Staging System

American Joint Committee on Cancer (AJCC) TNM Staging Classification for Carcinoma of the Stomach (8th ed, 2017)

- **Definition of Primary Tumor (T)**

T_x Primary tumor cannot be assessed

T_0 No evidence of primary tumor

Tis Carcinoma in situ: intraepithelial tumor without invasion of the lamina propria, high-grade dysplasia

T_1 Tumor invades lamina propria, muscularia mucosa or sub-mucosa

T_{1a} Tumor invades the lamina propria or muscularis mucosae

T_{1b} Tumor invades the sub-mucosa

T_2 Tumor invades the muscularis propria

T_3 Tumor penetrates the subserosal connective tissue without invasion of the visceral peritoneum or adjacent structures

T_4 Tumor invades the serosa (visceral peritoneum) or adjacent structures

T_{4a} Tumor invades the serosa (visceral peritoneum)

T_{4b} Tumor invades adjacent structures/organs

- **Definition of Regional Lymph Node (N)**

N_x Regional lymph node(s) cannot be assessed

N_0 No regional lymph node metastasis

N_1 Metastasis in one or two regional lymph nodes

N_2 Metastasis in three to six regional lymph nodes

N_3 Metastasis in seven or more regional lymph nodes

N_{3a} Metastasis in seven to 15 regional lymph nodes

N_{3b} Metastasis in 16 or more regional lymph nodes

- **Definition of Distant Metastasis (M)**

M_0 No distant metastasis

M_1 Distant metastasis

Post-Neoadjuvant Therapy (ypTNM)

When T is...	And N is...	And M is...	Then the stage group is...
T1	N0	M0	I
T2	N0	M0	I
T1	N1	M0	I
T3	N0	M0	II
T2	N1	M0	II
T1	N2	M0	II
T4a	N0	M0	II
T3	N1	M0	II
T2	N2	M0	II
T1	N3	M0	II
T4a	N1	M0	III
T3	N2	M0	III
T2	N3	M0	III
T4b	N0	M0	III
T4b	N1	M0	III
T4a	N2	M0	III
T3	N3	M0	III
T4b	N2	M0	III
T4b	N3	M0	III
T4a	N3	M0	III
AnyT	AnyN	MI	IV

Appendix 3 Surgical Manual

In this study, the basic purpose of surgery is curative resection. Curative resection is defined as follows:

- No distant metastasis.
- No infiltration to surrounding organs, or in case of infiltration, curative combined resection should be performed.
- No macroscopic residual cancer.
- D2 or greater resection is necessarily required.
- No infiltration of cancer cells on both margins from histopathology.

Selection of gastrectomy The standard surgical procedure for clinically node-positive (cN+) or T2-T4a tumors is either total or distal gastrectomy. Distal gastrectomy is selected when a satisfactory proximal resection margin (see below) can be obtained. When obtaining proximal resection margin is not possible, total gastrectomy is selected.

Resection margin A sufficient resection margin should be ensured when determining the resection line in gastrectomy with curative intent. Proximal margin of at least 3 cm is recommended for T2 or deeper tumors with an expansive growth pattern (types 1 and 2) and 5 cm for those with an infiltrative growth pattern (types 3 and 4). When these rules cannot be satisfied, it is advisable to examine the whole thickness of proximal resection margin by frozen section. For tumors invading the esophagus, resection margin >5 cm is not necessarily required, but frozen section examination of the resection line is preferable to ensure an R0 resection.

Definition of the D levels

- **Total gastrectomy**

D0: Lymphadenectomy less than D1.

D1: No. 1-7.

D1+: D1 + No. 8a, 9, 11p.

D2: D1 + No. 8a, 9, 11p, 11d, 12a.

For tumors invading the esophagus, resection of No.110 should be added to D1+, and resection of Nos. 19, 20, 110 and 111 to D2.

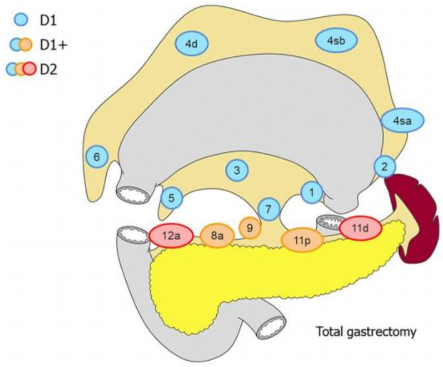

- **Distal gastrectomy**

D0: Lymphadenectomy less than D1.

D1: No. 1, 3, 4sb, 4d, 5, 6, 7.

D1+: D1 + No. 8a, 9.

D2: D1 + No. 8a, 9, 11p, 12a.

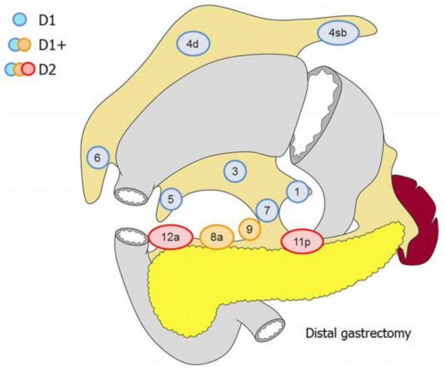

- **Proximal gastrectomy**

D0: Lymphadenectomy less than D1.

D1: No. 1, 2, 3a, 4sa, 4sb, 7.

D1+: D1 + No. 8a, 9, 11p.

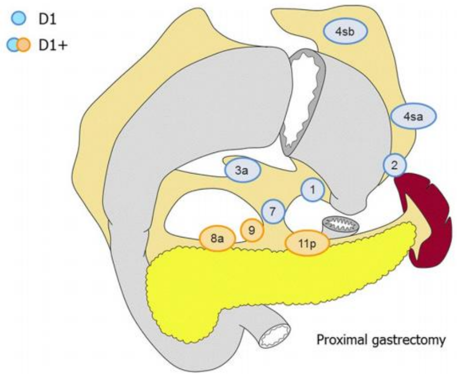

Appendix 4 Response Evaluation Criteria in Solid Tumors (RECIST), Version 1.1

Measurability of tumor at baseline

At baseline, tumor lesions/lymph nodes will be categorised measurable or non-measurable as follows:

- Measurable

Tumor lesions: Must be accurately measured in at least one dimension (longest diameter in the plane of measurement is to be recorded) with a minimum size of:

• 10 mm by CT scan (CT scan slice thickness no greater than 5 mm).

• 10 mm caliper measurement by clinical exam (lesions which cannot be accurately measured with calipers should be recorded as non-measurable).

• 20 mm by chest X-ray.

Malignant lymph nodes: To be considered pathologically enlarged and measurable, a lymph node must be ≥15 mm in short axis when assessed by CT scan (CT scan slice thickness recommended to be no greater than 5 mm). At baseline and in follow-up, only the short axis will be measured and followed.

- Non-measurable

All other lesions, including small lesions (longest diameter <10 mm or pathological lymph nodes with ≥10 to <15 mm short axis) as well as truly non-measurable lesions. Lesions considered truly non-measurable include: leptomeningeal disease, ascites, pleural or pericardial effusion, inflammatory breast disease, lymphangitic involvement of skin or lung, abdominal masses/abdominal organomegaly identified by physical exam that is not measurable by reproducible imaging techniques.

Response criteria

This section provides the definitions of the criteria used to determine objective tumour response for target lesions.

- **Evaluation of target lesions**

Complete Response (CR): Disappearance of all target lesions. Any pathological lymph nodes (whether target or non-target) must have reduction in short axis to <10 mm.

Partial Response (PR): At least a 30% decrease in the sum of diameters of target lesions, taking as reference the baseline sum diameters.

Progressive Disease (PD): At least a 20% increase in the sum of diameters of target lesions, taking as reference the smallest sum on study (this includes the baseline sum if that is the smallest on study). In addition to the relative increase of 20%, the sum must also demonstrate an absolute increase of at least 5 mm. (Note: the appearance of one or more new lesions is also considered progression).

Stable Disease (SD): Neither sufficient shrinkage to qualify for PR nor sufficient increase to qualify for PD, taking as reference the smallest sum diameters while on study。

- **Evaluation of non-target lesions**

This section provides the definitions of the criteria used to determine the tumour response for the group of non-target lesions.

While some non-target lesions may actually be measurable, they need not be measured and instead should be assessed only qualitatively at the time points specified in the protocol.

Complete Response (CR): Disappearance of all non-target lesions and normalisation of tumour marker level. All lymph nodes must be non-pathological in size (<10 mm short axis).

Non-CR/Non-PD: Persistence of one or more non-target lesion(s) and/or maintenance of tumour marker level above the normal limits.

Progressive Disease (PD): Unequivocal progression (see comments below) of existing non-target lesions. (Note: the appearance of one or more new lesions is also considered progression).

Appendix 5 ECOG Performance Status

According to the simplified performance status score scale developed by the ECOG, the patients' performance status can be classified into 6 levels, namely 0-5, as follows:

ECOG Performance Status

Grade	Characteristic
0	Fully active, able to carry on all pre-disease performance without restriction.
1	Restricted in physically strenuous activity but ambulatory and able to carry out work of a light or sedentary nature, e.g., light housework, office work.
2	Ambulatory and capable of all self-care but unable to carry out any work activities. Up and about more than 50% of waking hours.
3	Capable of only limited self-care, confined to bed or chair more than 50% of waking hours.
4	Completely disabled. Cannot carry on any self-care. In total, confined to bed or chair.
5	Dead.

Note: Patients at levels 3, 4 and 5 are generally considered to be unsuitable for surgical treatment or chemotherapy.

Appendix 6 Classification of Surgical Complications

Clavien-Dindo Classification

Grade	Characteristic
**Grade I**	Any deviation from the normal postoperative course without the need for pharmacological treatment or surgical, endoscopic, and radiological interventions. Allowed therapeutic regimens are: drugs as antiemetics, antipyretics, analgetics, diuretics, electrolytes, and physiotherapy. This grade also includes wound infections opened at the bedside.
**Grade II**	Requiring pharmacological treatment with drugs other than such allowed for grade I complications.
**Grade III**	Requiring surgical, endoscopic or radiological intervention<br>**Grade IIIa:** Intervention not under general anesthesia<br>**Grade IIIb:** Intervention under general anesthesia
**Grade IV**	Life-threatening complication requiring ICU management<br>**Grade IVa:** Single organ dysfunction (including dialysis)<br>**Grade IVb:** Multiorgan dysfunction
**Grade V**	Death of a patient.

Efficacy and Safety of Camrelizumab and Apatinib Combined with Nab-paclitaxel and S-1 as Neoadjuvant Treatment for Locally Advanced Gastric Cancer: A Multicenter Randomized Controlled Trial

SUMMARY OF CHANGES

Please note the statistical analysis plan is included in section 9.3 of both protocols.

Protocol Version History

Version Number	Version Date	Scientific and Substantive Revisions
v1.0	October 20, 2019	● Initial protocol
v1.1	December 15, 2019	● First Affiliated Hospital of Fujian Medical University, Fujian Provincial Cancer Hospital, First Affiliated Hospital of Xiamen University, and The First Hospital of Putian were removed from the Coordinating Institutions.<br>● The planned start date, planned enrollment completion date, and planned study end date were changed as January 1 2020, December 31 2021, and December 31 2024, respectively.
v1.2	February 25, 2020	● We supplemented the following contents in the Statistical Analysis Plan: The relationship between biomarkers (including but not limited in PD-L1 expression and MSI status) and the MPR rate will be presented for a subset of patients in the efficacy evaluable population who are available for these biomarkers.<br>● The planned start date, planned enrollment completion date, and planned study end date were changed as June 1 2020, June 1 2022, and June 1 2025, respectively.
v1.3	April 11, 2020	● We replaced the ITT set with the modified ITT set in efficacy analysis in the Statistical Analysis Plan.
