# Supplementary material for: Neoadjuvant camrelizumab and apatinib combined with chemotherapy versus chemotherapy alone for locally advanced gastric cancer: a multicenter randomized phase 2 trial
Source: Nat Commun. 2024 Jan 2;15:41. doi: 10.1038/s41467-023-44309-5 (PMC10762218; doi:10.1038/s41467-023-44309-5)
Supplement: Supplementary file 3 — Reporting Summary [file 41467_2023_44309_MOESM3_ESM.pdf]

nature portfolio

Corresponding author(s): Changming Huang, Ping Li

Last updated by author(s): Nov 22, 2023

Reporting Summary

Nature Portfolio wishes to improve the reproducibility of the work that we publish. This form provides structure for consistency and transparency in reporting. For further information on Nature Portfolio policies, see our Editorial Policies and the Editorial Policy Checklist.

Statistics

For all statistical analyses, confirm that the following items are present in the figure legend, table legend, main text, or Methods section.

n/a	Confirmed	
☐	☒	The exact sample size (*n*) for each experimental group/condition, given as a discrete number and unit of measurement
☐	☒	A statement on whether measurements were taken from distinct samples or whether the same sample was measured repeatedly
☐	☒	The statistical test(s) used AND whether they are one- or two-sided<br>*Only common tests should be described solely by name; describe more complex techniques in the Methods section.*
☐	☒	A description of all covariates tested
☐	☒	A description of any assumptions or corrections, such as tests of normality and adjustment for multiple comparisons
☐	☒	A full description of the statistical parameters including central tendency (e.g. means) or other basic estimates (e.g. regression coefficient) AND variation (e.g. standard deviation) or associated estimates of uncertainty (e.g. confidence intervals)
☐	☒	For null hypothesis testing, the test statistic (e.g. *F*, *t*, *r*) with confidence intervals, effect sizes, degrees of freedom and *P* value noted<br>*Give P values as exact values whenever suitable.*
☒	☐	For Bayesian analysis, information on the choice of priors and Markov chain Monte Carlo settings
☒	☐	For hierarchical and complex designs, identification of the appropriate level for tests and full reporting of outcomes
☒	☐	Estimates of effect sizes (e.g. Cohen's *d*, Pearson's *r*), indicating how they were calculated

Our web collection on statistics for biologists contains articles on many of the points above.

Software and code

Policy information about availability of computer code

Data collection	All data were collected with Microsoft Excel.
Data analysis	All statistical analyses were conducted with SPSS statistical software (version 21.0; SPSS Inc.) and R software (version 4.1.2; R Foundation for Statistical Computing).

For manuscripts utilizing custom algorithms or software that are central to the research but not yet described in published literature, software must be made available to editors and reviewers. We strongly encourage code deposition in a community repository (e.g. GitHub). See the Nature Portfolio guidelines for submitting code & software for further information.

Data

Policy information about availability of data

All manuscripts must include a data availability statement. This statement should provide the following information, where applicable:

- Accession codes, unique identifiers, or web links for publicly available datasets
- A description of any restrictions on data availability
- For clinical datasets or third party data, please ensure that the statement adheres to our policy

The data supporting the findings in this study are available under controlled access due to data privacy laws related to patient consent for data sharing and the data should be used for research purposes only. All the original clinical data will be made available on request from the corresponding author (Huang CM) at any time in a de-identified manner. The remaining data are available within the Article, Supplementary Information, or Source Data file. Request for data sharing will be handled in line with the data access and sharing policy of Fujian Medical University Union Hospital. The original study protocol is available in the Supplementary Information

as a Supplementary Note. Source data are provided with this paper.

Research involving human participants, their data, or biological material

Policy information about studies with human participants or human data. See also policy information about sex, gender (identity/presentation), and sexual orientation and race, ethnicity and racism.

Reporting on sex and gender	I. Findings are not applied to only one sex.<br>II. Sex was not considered in study design.<br>III. Sex was determined based on self-reporting.<br>IV. Sex information has been collected, and written consent has been obtained for sharing of individual-level data.<br>V. Of all, 77 were males and 27 were females.<br>VI. Subgroup analysis according to sex was performed in this study.
Reporting on race, ethnicity, or other socially relevant groupings	Socially constructed or socially relevant categorization variables were not used in this study.
Population characteristics	I. Age older than 18 and younger than 75 years<br>II. Primary gastric adenocarcinoma confirmed pathologically by endoscopic biopsy<br>III. Clinical stage T2-4aN+M0 disease as assessed by CT/MRI, PET-CT, and laparoscopy, if feasible<br>IV. At least one measurable lesion according to the RECIST, version 1.1<br>V. ECOG performance status of 0 or 1<br>VI. Life expectancy of at least 12 weeks<br>VII. Acceptable bone marrow, hepatic, and renal function
Recruitment	This randomized controlled trial was conducted at 5 Chinese centers between June 18, 2020 and March 31, 2022. Any gastric cancer patient who was admitted at the participant centers, and met the criteria for inclusion was considered for recruitment, thus we confirm that there was no selection bias during recruitment. Patients were screened by the trained clinicians at the participant centers and the principal investigators were responsible for the evaluation of pretreatment assessment and deciding for enrollment. All the participants provided written informed consent before screening.
Ethics oversight	The study protocol and all amendments were approved by the institutional review board of the Fujian Medical University Union Hospital, Second Affiliated Hospital of Fujian Medical University, Zhongshan Hospital of Xiamen University, Zhangzhou Municipal Hospital of Fujian Province, and The Affiliated Hospital of Putian University. All patients provided written informed consent. The study was performed in accordance with the Declaration of Helsinki and Good Clinical Practice guidelines.

Note that full information on the approval of the study protocol must also be provided in the manuscript.

Field-specific reporting

Please select the one below that is the best fit for your research. If you are not sure, read the appropriate sections before making your selection.

☒ Life sciences ☐ Behavioural & social sciences ☐ Ecological, evolutionary & environmental sciences

For a reference copy of the document with all sections, see nature.com/documents/nr-reporting-summary-flat.pdf

Life sciences study design

All studies must disclose on these points even when the disclosure is negative.

Sample size	Based on the assumption of MPR rates of 15% in the SAP group and 35% in the CA-SAP group, a sample size of 53 patients per group was required to detect improvement with 80% power and a one-sided alpha level of 0.1 (Fisher's exact test), including a 5% dropout rate.
Data exclusions	No data were excluded from the analyses.
Replication	To verify the reproducibility of results, all the described results were tested for replication by two independent statisticians. In addition, the expression and score of PD-L1 expression and MSI status were evaluated by the same two pathologists according to a unified scoring standard to exclude the result bias caused by measurement errors.
Randomization	A blinded statistician performed randomization with a list of randomly ordered treatment identifiers generated by SAS software, version 9.2 (SAS Institute). The randomized sequence was created for 1:1 allocation of 106 cases, 53 cases in each group, and was concealed from the investigators who screened and enrolled participants. The assignment was made by telephone contact or text messages after the patient met the eligibility criteria and signed the informed consent form.
Blinding	Patients and caregivers were not blinded to the treatment received. Outcome assessment for the primary endpoint was performed by two blinded pathologists. All statistical analyses were also performed by a blinded investigator.

Reporting for specific materials, systems and methods

We require information from authors about some types of materials, experimental systems and methods used in many studies. Here, indicate whether each material, system or method listed is relevant to your study. If you are not sure if a list item applies to your research, read the appropriate section before selecting a response.

Materials & experimental systems

n/a	Involved in the study
☐	☒ Antibodies
☒	☐ Eukaryotic cell lines
☒	☐ Palaeontology and archaeology
☒	☐ Animals and other organisms
☐	☒ Clinical data
☒	☐ Dual use research of concern
☒	☐ Plants

Methods

n/a	Involved in the study
☒	☐ ChIP-seq
☒	☐ Flow cytometry
☒	☐ MRI-based neuroimaging

Antibodies

Antibodies used

PD-L1 expression was measured with the Ventana PD-L1 (SP263) immunohistochemistry assay. MSI status was measured with MLH1 (ab92312, Abcam, 1 : 250), MSH2 (ab52266, Abcam, 1 : 250), MSH6 (ab92471, Abcam, 1 : 250), PMS2 (ab110638, Abcam, 1 : 250) immunohistochemical staining on the tissue.

Validation

The validation statements can be found on the manufacturer's website.

Clinical data

Policy information about clinical studies

All manuscripts should comply with the ICMJEguidelines for publication of clinical research and a completedCONSORT checklist must be included with all submissions.

Clinical trial registration

ClinicalTrials.gov Identifier: NCT04195828

Study protocol

Study protocol has been uploaded.

Data collection

The data was collected by investigators at each center. Demographic and clinical details were obtained at randomization. The primary endpoint was centrally evaluated by two blinded pathologists according to a standardized procedure manual. The data collected between June 2020 and May 2022.

Outcomes

The primary endpoint was the MPR rate, defined as the proportion of patients with <10% residual tumor cells in resection specimens. The MPR rate was evaluated centrally in a standardized procedure manual.
Secondary endpoints included the pCR rate (defined as no residual tumor cells in resection specimens assessed by blinded pathologists), R0 resection rate (defined as complete resection without macroscopic or microscopic residual disease assessed by surgeons and blinded pathologists), radiologic response (evaluated using RECIST version 1.1 by local radiologists), safety (evaluated according to the Common Terminology Criteria for Adverse Events, version 5.0 and Clavien–Dindo classification), and survival (collected by the principle investigators).
